# Development of whole-genome multiplex assays and construction of an integrated genetic map using SSR markers in Senegalese sole

**DOI:** 10.1038/s41598-020-78397-w

**Published:** 2020-12-14

**Authors:** Israel Guerrero-Cózar, Cathaysa Perez-Garcia, Hicham Benzekri, J. J. Sánchez, Pedro Seoane, Fernando Cruz, Marta Gut, Maria Jesus Zamorano, M. Gonzalo Claros, Manuel Manchado

**Affiliations:** 1 IFAPA Centro El Toruño, Junta de Andalucía, Camino Tiro Pichón s/n, 11500 El Puerto de Santa María, Cádiz Spain; 2 Aquaculture Research Group (GIA), IU-ECOAQUA, Universidad de Las Palmas de Gran Canaria, Crta. Taliarte s/n, 35214 Telde, Spain; 3 Department of Molecular Biology and Biochemistry, Universidad de Málaga, 29071 Málaga, Spain; 4 Instituto Nacional de Toxicología Y Ciencias Forenses (INT), La Cuesta, La Laguna, 38320 Sta. Cruz de Tenerife, Spain; 5 CNAG-CRG, Centre for Genomic Regulation (CRG), The Barcelona Institute of Science and Technology (BIST), Baldiri Reixac 4, 08028 Barcelona, Spain; 6 CIBER de Enfermedades Raras (CIBERER), 29071 Málaga, Spain; 7 Institute of Biomedical Research in Málaga (IBIMA), IBIMA-RARE, 29010 Málaga, Spain; 8 Instituto de Hortofruticultura Subtropical Y Mediterránea (IHSM-UMA-CSIC), 29010 Málaga, Spain; 9 “Crecimiento Azul”, Centro IFAPA El Toruño, Unidad Asociada Al CSIC”, El Puerto de Sta María, Spain

**Keywords:** Animal breeding, Genetic markers

## Abstract

The Senegalese sole (*Solea senegalensis*) is an economically important flatfish species. In this study, a genome draft was analyzed to identify microsatellite (SSR) markers for whole-genome genotyping. A subset of 224 contigs containing SSRs were preselected and validated by using a de novo female hybrid assembly. Overall, the SSR density in the genome was 886.7 markers per megabase of genomic sequences and the dinucleotide motif was the most abundant (52.4%). In silico comparison identified a set of 108 SSRs (with di-, tetra- or pentanucleotide motifs) widely distributed in the genome and suitable for primer design. A total of 106 markers were structured in thirteen multiplex PCR assays (with up to 10-plex) and the amplification conditions were optimized with a high-quality score. Main genetic diversity statistics and genotyping reliability were assessed. A subset of 40 high polymorphic markers were selected to optimize four supermultiplex PCRs (with up to 11-plex) for pedigree analysis. Theoretical exclusion probabilities and real parentage allocation tests using parent–offspring information confirmed their robustness and effectiveness for parental assignment. These new SSR markers were combined with previously published SSRs (in total 229 makers) to construct a new and improved integrated genetic map containing 21 linkage groups that matched with the expected number of chromosomes. Synteny analysis with respect to C. *semilaevis* provided new clues on chromosome evolution in flatfish and the formation of metacentric and submetacentric chromosomes in Senegalese sole.

## Introduction

Genomes are an essential source of markers required for ecological studies, breeding programs, traceability or functional studies. In the last years, the genomes of some commercially important flatfish belonging to the Cynoglossidae, Scophthalmidae, and Paralichthydae families were published indicating that overall, they are small and highly compact with sizes ranging between 470 and 584 Mb^1–4^. These genomes have contributed to a better understanding of chromosome evolution in flatfish^5^, sex determination^2^ and the identification of mechanisms controlling metamorphosis^4^ and growth performance^6^ with impact in aquaculture and stock population management. In Senegalese sole (*Solea senegalensis*), a preliminary draft of 600.3 Mb that fully covered the tongue sole (*Cynoglossus semilaevis*) genome was assembled^7,8^. Although this assembly was still a bit fragmented (N50 of 85 kb), it became an useful tool to understand hybridization and introgression between *S. senegalensis* and *S. aegyptiaca*^9^ and for synteny analysis^8,10,11^. Nevertheless, an improvement of scaffolding and chromosome architecture is required for association studies, gene mapping and comparative genomics.

Genetic linkage maps and physical genomes provide complementary information that can be useful for the refinement of genome assemblies, the identification of genes associated with QTLs and cross-species synteny analysis^12,13^. In Senegalese sole, a low-density genetic linkage map constructed using three gynogenetic families and 129 microsatellites (also known as simple sequence repeats, SSRs) markers was described^14^. This map contained 27 linkage groups (LG) with an average density of 4.7 markers per LG that it was still a bit far away from the 21 chromosomes expected in *S. senegalensis*. Comparative synteny mapped these LGs through most of the chromosomes (except three) of *C. semilaevis* suggesting that some chromosome rearrangements could have occurred during evolution of these species^8^. Moreover, an integrated map using BAC clones and repetitive DNA families was developed using multiple fluorescence in situ hybridization that comprised 64 BACs mapped through all genome except in the submetacentric chromosome five^15^. Although Senegalese sole has not morphologically heteromorphic sex chromosomes, the largest metacentric chromosome was proposed as a proto-sex chromosome originated from the fusion of two acrocentric chromosomes during flatfish evolution^12,16^.

Even though SNP markers have attracted the attention of researchers in the last years to construct high-density genetic linkage maps and for genetic association studies^17^, the SSR markers still remain as highly popular markers due to their high variability, reproducibility, and their codominant inheritance^18,19^. To maximize the use of SSR markers, whole-genome genotyping using SSR-based multiplex PCRs have become the most suitable strategy to save costs, labour time and reduce data processing. This methodological approach can make feasible the implementation in small- to medium-sized laboratories since it requires basic equipment with comparable results between laboratories^20,21^. These whole-genome multiplex PCRs have been successfully applied to pedigree reconstruction in genetic breeding programs and QTLs identification^22–25^. However, *loci* multiplexing requires a tailor-made design of primers to be combined and amplified simultaneously avoiding primer dimer and preventing the overlapping of allelic ranges in those markers labelled with the same fluorophore colour. Hence, in silico analysis of genome SSR information followed by experimental validation of multiplex PCR assays is required.

Senegalese sole genome and transcriptome are rich mainly in SSRs with dinucleotide motif representing ~ 60% of total SSRs, tetranucleotides only 5.2% and pentanucleotides 2.4%^15,26^. Although SSRs with dinucleotide motifs have a higher allelic diversity than those with larger motifs, these latter are less prone to artefacts such as allelic dropout and stutters. Hence, scoring accuracy is very high reducing genotyping errors and making feasible data automation^27,28^. Genome analysis provides enough information for in silico analysis to select and combine high polymorphic SSR markers while they maintain an reliable and robust scoring for multiplex PCRs. The aim of this study was to: (1) provide de novo improved assembly of a female Senegalese sole based on long and short reads; (2) identify tetra- or pentanucleotide SSRs in silico and carry out a flatfish cross-species comparison to design whole-genome Multiplex PCRs; (3) validate all SSR *loci*, structure in multiplex PCRs according to allelic ranges (with up to 11-plex amplification) and optimize amplification conditions for whole genome mapping; (4) design supermultiplex PCRs containing the most polymorphic *loci* to sustain breeding genetic programs in this species in which offspring is communally reared; and (5) integrate SSR markers available in Senegalese sole in a genetic linkage map and carry out a synteny analysis with the flatfish *C. semilaevis* to understand chromosome evolution.

## Methods

### Genome sequencing, assembly and characterization

SSR identification was carried out by in silico analysis of a previously published female genome based on Illumina short-reads^7,8^. Both the contig (named as assembly_51k according to k-mer used) and the scaffolded (named as 85 k genome according to N50) assemblies were used.

To increase the reliability of predicted SSR flanking regions, genome positioning and map distribution, a de novo female hybrid genome was also assembled using short and long reads. High molecular weight DNA was prepared from heparinized whole blood using the MagAttract HMW DNA kit (Qiagen). Main figures of Oxford nanopore Technology (ONT) (female code H2074515) and Illumina paired-end (PE300) reads (female code H150612; Bioproject PRJNA643826) are depicted in Table 1. Sequencing was carried out at the National Center for Genomic Analysis (CNAG, Barcelona, Spain). For the hybrid assembly, libraries libraries were pre-processed to remove contaminants and low-quality sequences. Briefly, the Illumina PE300 library was screened using Kraken (v0.10.5-beta)^29^ and contaminants filtered out with the gem-mapper^30^ (with ≤ 2% mismatches). In the case of ONT, data were base-called with Albacore v2.0.2 and reads meeting the following criteria were filtered out: base quality per read Q < 7, match to the control Sequence (lambda phage 3.5 kb), length less than 1 kb, or more than 40% low complexity sequence. Finally, POMOXIS v0.1.0 (https://github.com/nanoporetech/pomoxis) and Racon^31^ via all-vs-all alignment with minimap2^32^ were used to correct the reads before assembly. The hybrid genome assembly (named as LR-hybrid female genome) was carried using MaSuRCA v3.2.3^33,34^ to construct mega-reads that were finally assembled with CABOG v6.2^35^. Completeness was determined using Benchmarking Universal Single-Copy Orthologs (BUSCO, v3.0.2)^36,37^ containing 4854 single-copy orthologs from actinopterygii_odb9. Genome scaffolds are available at Claros et al.^38^.

**Table 1:** Summary of input datasets for Illumina (PE300) and Oxford Nanopore Technologies (ONT) reads for LR hybrid female assembly.

Library	Read length N50 (bp)	Fragment length (bp)	Total reads	Yield (Gb)	Error r1 (%)	Error r2 (%)	Sequencing coverage^b^
PE300	101	330	1,005,526	101.56	0.29	0.62	142.24
ONT 1DSQ	8203	–	64,016	0.40	6.7		0.56
ONT MinION^a^	10,802	–	1,311,044	9.38	17.6	–	12.57

^a^Information corresponding to the filtered 1D and 1D2 reads produced by five MinION runs. Error rate estimated as sum of mismatched, inserted bases and deleted bases divided by length of alignment of Oxford Nanopore Technologies (ONT) reads to the control sequence

^b^Coverage estimates are calculated assuming a genome size of 714 Mb (C-value of *Solea solea*).

### SSR screening, primer design and in silico genome mapping

SSR screening on the genomes was carried out using MISA (Microsatellite identification tool) and the parameters were those previously described^39^. A total of 224 contigs from the the 85 k genome larger than 20 kb and containing several SSRs were preselected and positioned onto the *C. semilaevis* genome by local blast analysis (Supplementary Table S1 tab "Preselected_contigs"). Moreover, unigenes from Senegalese sole transcriptome^26^ were positioned within each contig to identify gene content and sysnteny with *C. semilaevis*. A final set of putative 113 tetra- or pentanucleotide SSRs located in contigs from different chromosomes or separated at least 1 Mb apart within the same chromosome were selected (Supplementary Table S1 tab "Selected contigs"). To validate chromosome positioning, these selected contigs were further mapped onto the LR-hybrid female genome and the scaffolds blasted onto *C. semilaevis* chromosomes.

The criteria followed for primer design were those previously described for multiplex PCR reactions^21,40^. Primer sequences in each multiplex PCR assay and fluorophore labelling are depicted in Supplementary Table S2. The range of amplicon sizes oscillated between 70 and 300 base pairs (bp). The primer quality and amplicon specificity were assessed by mapping sequences onto the de novo LR-hybrid female genome (Supplementary Table S2, tab "PrimerMappingSSR"). A quality scale was established as follows: (1) high-specific (H–S) when they yielded a single specific amplicon and they mapped just in one position in the genome; (2) specific (S) when they yielded a single specific amplicon but at least one of the primers mapped between 2–10 (S* 2), 11–100 (S**) or > 100 (S***) positions in the genome; (3) multiple (M) when the primers amplified different regions in the genome; and (4) no amplification (NA) when no amplicon could be predicted or the amplicon was larger than 300 bp. A similar strategy was pursued to evaluate the quality of the primers published by Molina-Luzon, et al.^14^ (Supplementary Table S2, tab "PrimerMappingLuzon").

### Fish samples and DNA isolation

To characterize the SSR markers, wild specimens of Senegalese sole captured in the Gulf of Cádiz (Spain) and incorporated to the aquaculture broodstocks of the company CUPIMAR (San Fernando, Cádiz, Spain) and IFAPA center El Toruño (El Puerto de Santa María, Cádiz, Spain) were used. Animals were sampled for blood (~ 0.5 ml) by puncturing in the caudal vein using a heparinized syringe, added heparin (100 mU) and kept at − 20 °C until use. Overall, the whole set of animals used in this study was 150 (79 breeders from CUPIMAR and 71 from IFAPA). To optimize the multiplex PCR assays, the 71 animals from IFAPA's broodstock structured in four tanks (n = 6, 21, 22, and 22 fish) were used. As we carried out several tests to adjust the primer conditions and validate amplifications, some samples were run out and the total individuals finally analyzed in each multiplex PCR assay was slighlty different (althout the four tanks were represented in all assays) and specifically indicated in each case. To validate the supermultiplex PCR assays and carry out the simulations, fish from CUPIMAR (n = 79 distributed in four tanks) and IFAPA (n = 13) was used.

Total DNA from heparinized blood (~ 25 µl) was isolated using Isolate II Genomic DNA Kit (Bioline). DNA samples were treated with RNase A (Bioline) following the manufacture’s protocol. DNA was quantified spectrophotometrically using the Nanodrop ND-8000. Each microsatellite marker was tested in singlepex PCR to confirm amplification. PCR reactions were carried out in a 12.5 µl final volume containing 40 ng of DNA, 300 nM each of specific forward and reverse primers, and 6.25 µl of Platinum Multiplex PCR Master Mix, 2 × (Thermofisher Scientific). The amplification protocol consisted of an initial denaturation at 95 °C for 10 min, followed by 30 cycles of 95 °C for 20 s, 59 °C for 1 min and 72 °C for 2 min, with a final extension of 72 °C for 10 min. PCR products were separated by capillary electrophoresis in an ABI3130 Genetic Analyzer (Applied Biosystems). Raw data obtained by capillary electrophoresis were transformed into allelic sizes using the GeneMapper v3.8 software (Thermofisher Scientific).

### Multiplex PCRs optimization

SSRs were initially distributed in thirteen multiplex PCR assays (ranging 6 to 10-plex amplification) (Supplementary Table S2 tab "InitialMultiplexDesign". However, when markers were tested in singleplex, three of them did not amplify (SSeneg12220, SSeneg13367 and SSeneg3342) and two (SSeneg977 and SSeneg398) amplified a multipeak patterning and they were removed from the original sets. Moreover, SSeneg3502 and SSeneg106 markers were excluded from the mutiplex PCRs due to overlapping allelic range with other markers or a low amplification efficiency. The final thirteen multiplex PCR sets (named from A to M) are indicated in Supplementary Table S2 (tab "FinalMultiplex"). All Multiplex PCRs were performed in a final volume of 12.5 μl containing 1 × Platinum Multiplex PCR Master Mix, 40 ng of template DNA and the primer concentrations indicated in Supplementary Table S2 (tab "Primer amounts") that were optimized to balance the fluorescent signal intensity. The PCR program is the same indicated above and the final electropherograms obtained for each Multiplex set are shown in Supplementary Fig. S1.

To validate the robustness of the whole-genome multiplex PCRs, an independent lab (University of Las Palmas de Gran Canaria, Spain) analyzed a subset of DNA samples from IFAPA's broodstock (total n = 60). The specific number of samples analyzed for each *locus* in the multiplex PCRs is indicated in Supplementary Table S3. The amplification conditions were similar to those indicated above except that Platinum Multiplex PCR Master Mix was replaced by KAPA2G Fast Multiplex PCR Kit (Kappa Biosystems_Sigma Aldrich). Electropherograms were analyzed using Genemapper (v.3.8) software (Applied Biosystems) and a kit of bin set was created for each multiplex PCR. A protocol for evaluation of genotyping reliability and *loci* scoring was performed^21^. Briefly, the rate of errors or potential errors for each marker were determined after identifying ambiguous or unambiguous genotypes in the samples. The main genotyping errors were classified as inadequate peak heights out of optimal ratio (600–3000 relative fluorescent units), unclear banding pattern or intermediate alleles that could not be read automatically using the bin set.

In order to design genotyping tools for parentage assignments in genetic breeding programs, a set of 40 SSR markers with the highest variability according to the polymorphic information content (PIC) was selected and rearranged in four new supermultiplex (SM) assays considering the fluorescent labelling and the allelic range (named as SMA, SMB, SMC and SMD). PCR amplification conditions were those described above and the primer cocktails optimized to balance peak signals are indicated in Supplementary Table S2 Tab "Primer amounts".

### Data analysis

Genetic diversity parameters (number of alleles (k)), observed (Ho) and expected (He) heterozygosities, allelic range, non-exclusion probabilities for pair parent (NE-PP) and null allele frequency were estimated using Cervus *v*3.0.3^41^. The Hardy–Weinberg equilibrium (HW) at each locus was tested based on χ^2^ tests using GenAlEx *v*6.502 software^42^. The test for null allele presence was performed using Micro-checker v2.2.3^43^. Parentage assignment was performed in PARFEX v1.0 using exclusion approach^44^. This package was further used to calculate the minimum marker set required for optimal parentage using the given data set. Markers were ranked according to PIC information and exclusion probability. In the case of SMA, a total of n = 92 specimens (48 females and 44 males; see "Fish samples" section) were analyzed. As the number of sole breeders in each tank oscillated between 13 and 25 specimens, simulations for supermultiplex SMB, SMC and SMD were carried out using a subset of animals (n = 15; 8 females and 7 males).

To construct the integrated SSR genetic map, the 108 SSR markers of this study and 121 out of 129 SSRs of the low density genetic linkage map available in Senegalese sole^14^ were positioned in the LR-hybrid female genome by local megablast analysis. Primers from eight markers in the previous map were excluded due to low quality mapping rates (Supplementary Table S2 tab "PrimerMappingLuzon"). Later, all scaffolds were anchored to the 21 linkage groups (LG) of a high-density SNP genetic linkage map generated using ddRAD from five full-sib families. Data about families, SNPs and full procedure to construct the SNP-based genetic linkage map will be published elsewhere. The relative genetic distances between makers were obtained from the anchored physical map and the integrated map was drawn using the software linkagemapview^45^. For macrosynteny comparison, scaffolds bearing the SSRs were blasted onto the *C. semilaevis* chromosomes and positions compared to identify chromosomal rearrangements.

### Compliance with ethical standards

All procedures were performed in accordance with Spanish national (RD 53/2013) and European Union legislation for animal care and experimentation (Directive 86\609\EU) and authorized by the Bioethics and Animal Welfare Committee of IFAPA and given the registration number 10/06/2016/101.

## Results

### Identification of SSRs for multiplex design and assessment of their genome distribution

SSR markers were identified by in silico analysis of repetitive motifs in the 85 k genome^7^ based on Illumina short-reads. A first search for SSR markers selected a set of 224 contigs bigger than 20 kb and putatively located in different chromosomes or separated at least 1 Mb apart in the same chromosome. Average size of selected contigs was 118.7 kb and a cross-species comparison with the genome of the flatfish *C. semilaevis* confirmed that they were widely distributed in all chromosomes (between 6 and 17 contigs by chromosome; Supplementary Table S1 tab "Preselection"). The average number of SSR markers in each contig was 14.6, 5.3, 4.3 and 2.3 for di-, tri- tetra- and pentanucleotide repeat motifs, respectively. Using as reference this information, a subset of 113 contigs putatively distributed through the genome (minimum 5 scaffolds by chromosome) containing SSRs with tetra- or pentanucleotide repeat motifs was selected (Supplementary Table S1 "Selected_contigs"). The final set of SSRs selected for primer design included 103 tetranucleotides, 5 pentanucleotides and 5 compound markers containing at least two tetranucleotide SSRs separated by a spacer (Supplementary Table S2 tab "InitialMultiplexDesign"). Overall, GATA was the most abundant repeat motif in the selected markers (30 SSRs).

To assess the conservation of SSR flanking regions and the expected amplicon sizes as indicator of SSR quality for primer design, a de novo assembly based on Nanopore long-reads corrected with Illumina reads was used (LR-hybrid female genome). Raw sequencing data are indicated in Table 1. Expected coverage was 141 × for Illumina PE300 library and 13.5 × for Nanopore reads. The new assembly resulted in 6,482 contigs and 5,748 scaffolds with a total length of 607,976,531 bp and scaffold N50 of 340 kb. The estimated gene integrity was 96.2%. Overall, the marker density was 886.7 SSRs per megabase (Mb) and the dinucleotide repeats were the most abundant (52.4%) followed by tri- (12.5%), tetra- (4.0%) and pentanucleotides (1.1%) (Supplementary Table S1, tab "SSR_genome"). The C/A motif represented the 75% of dinucleotide repeats. To assess the quality of 113 selected markers, all designed primers were mapped onto the scaffolds of LR-hybrid female genome and classified into four categories (high-specific (H–S), specific (S), multiple, (M) and no amplification (NA)) according to locus-specificity, predicted amplification success and amplicon size (Supplementary Table S2, tab "PrimerMappingSSR"). Primers of 74 markers mapped specifically in just one position and generated locus-specific PCR amplicons of expected size similiar to 85 k genome, 34 markers had one primer of the pair with more than one mapping through the genome although the primer pair generated a locus-specific PCR product of expected size, 2 markers were not locus-specific and 3 markers failed to provide a PCR product due to amplicon size larger than expected or mapping on different scaffolds (Supplementary Table S2 tab "PrimerMappingSSR"). After assessment primer quality, 108 markers were finally selected and arranged in multiplex PCRs. The wide distribution through the genome was validated by mapping scaffolds of the 85 k and LR-hybrid female genomes onto the *C. semilaevis* chromosomes (Supplementary Table S1 and Table S2). Mapping results were highly consistent between assemblies showing only some conflicts for those contigs (only13) located in the sexual chromosomes (Z and W) of *C. semilaevis* that are absent in sole.

### Whole-genome multiplex assays and genetic parameters

All SSR primers were designed to be amplified under similar conditions and hence they could be combined and ready for rearrangement between multiplex PCR assays depending on the labelling and allelic range. Before optimizing the multiplex reactions, all markers were tested in singleplex under the same amplification conditions.

The expected range of amplicon sizes for the complete set of SSR markers oscillated between 84 and 341 bp. Depending on the fluorescent labelling and the expected amplicon sizes, the 108 SSRs were distributed into 13 multiplex PCR assays (ranging from 6- and 10-plex) (Supplementary Table S2, tab "InitialMultiplexDesign"). After amplifying markers in fish samples, some of them had to be rearranged in other multiplex PCRs due to allelic range overlapping or low amplification efficiency in the assays and two markers (SSeneg3502 and SSeneg106) could not be combined in any way and they were excluded. Hence, the final design comprised 106 SSR markers amplified in thirteen multiplex PCRs (from 6 to 10-plex) (Supplementary Table S2 tab "FinalMultiplex"). Electropherograms obtained for each PCR multiplex assay and markers are shown in Supplementary Fig. S1.

**Figure 1:**
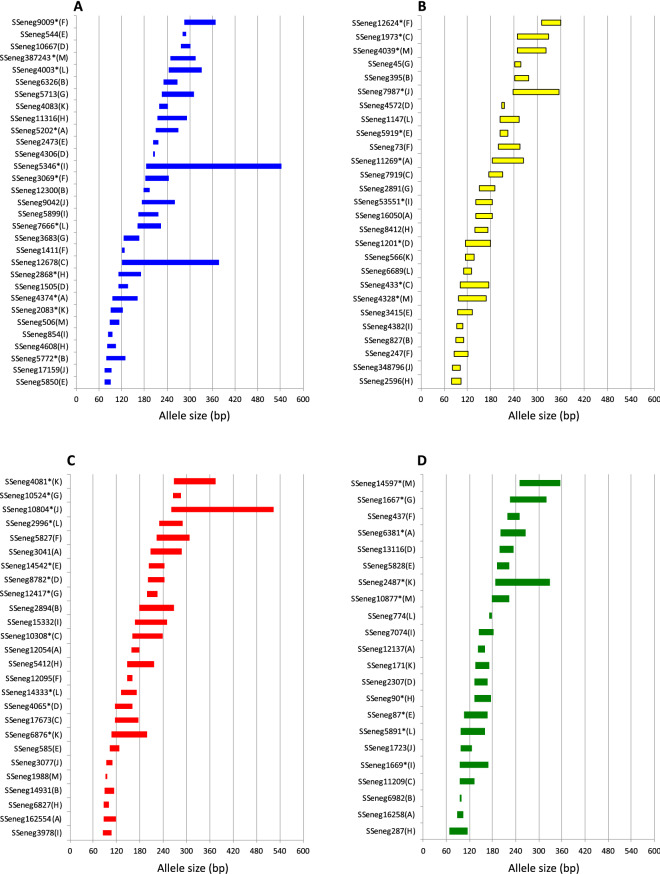
Allelic ranges of the 106 SSRs analysed in this study by fluorescence labelling (**A**–**D**). The name of the multiplex PCRs in which each marker is included is indicated between brackets. The asterisk indicates that the marker was selected to be included in the supermultiplex PCRs.

Main genetic parameters associated with each marker are depicted in Table 2. For each multiplex, between 44 and 71 specimens were analyzed. The number of alleles ranged between 2 and 43 by *loci*. Moreover, 89 SSR markers were experimentally confirmed as tetranucleotide and 5 as pentanucleotide after analysing the repetition patterns in genotyped samples. However, 13 SSR markers followed an allelic series compatible with a dinucleotide repeat motif. A total of 34 markers deviated from HW. Micro-checker results identified 24 markers with a possible presence of null alleles that in most of the cases deviated from HW. The allelic range of *loci* sorted by fluorescence labelling are depicted in Fig. 1. To test the robustness of the amplification and test the genetic variation of the markers, the thirteen PCR multiplex assays were run by an independent laboratory (ULPGC). Data comparison confirmed the genetic variability parameters, feasibility to amplify and consistent scoring of markers. Only 17 markers deviated from HW (Supplementary Table S3). *Loci* quality scoring identified 11 markers with a bit stuttering, 4 markers allele dropout and only two intermediate alleles but all of them could be successfully read.

**Table 2:** Genetic diversity estimates of 106 by multiplex PCRs (A-M).

**MultiplexA**												
Locus	L	Motif	N	k	Range	Ho	He	PIC	NE-PP	F(N)	HW	NA^&^
SSeneg4374	B	Tetra	61	10	96–162	0.53	0.72	0.69	0.28	0.16	ns	ns
SSeneg5202	B	Tetra	63	16	210–270	0.89	0.88	0.86	0.09	− 0.01	ns	ns
SSeneg16258	G	Tetra	63	4	88–104	0.48	0.51	0.47	0.54	0.02	ns	ns
SSeneg12137	G	Tetra	63	7	141–159	0.62	0.64	0.61	0.37	0.00	ns	ns
SSeneg6381	G	Tetra	63	18	200–266	0.71	0.87	0.85	0.10	0.10	(*)	Yes
SSeneg16050	Y	Tetra	63	10	142–184	0.49	0.62	0.59	0.38	0.12	ns	Yes
SSeneg11269	Y	Di	63	33	183–263	0.95	0.96	0.95	0.02	0.00	ns	ns
SSeneg162554	R	Tetra	63	9	86–118	0.89	0.80	0.76	0.21	− 0.06	ns	ns
SSeneg12054	R	Tetra	63	6	159–179	0.78	0.75	0.70	0.29	− 0.03	ns	ns
SSeneg3041	R	Tetra	63	14	207–287	0.52	0.78	0.75	0.22	0.19	*	Yes
**MultiplexB**												
SSeneg5772	B	Tetra	51	11	80–130	0.77	0.81	0.78	0.19	0.03	(*)	ns
SSeneg12300	B	Tetra	51	5	177–193	0.67	0.61	0.53	0.51	− 0.06	ns	ns
SSeneg6326	B	Tetra	51	9	231–267	0.82	0.80	0.77	0.19	− 0.02	ns	ns
SSeneg6982	G	Penta	51	2	94–100	0.28	0.27	0.23	0.81	− 0.02	ns	ns
SSeneg827	Y	Tetra	51	6	91–111	0.55	0.63	0.58	0.41	0.07	ns	ns
SSeneg395	Y	Penta	51	8	241–276	0.80	0.82	0.79	0.18	0.01	ns	ns
SSeneg14931	R	Tetra	51	7	89–113	0.69	0.77	0.73	0.26	0.06	ns	ns
SSeneg2894	R	Tetra	51	7	178–268	0.43	0.70	0.64	0.37	0.24	(*)	Yes
**MultiplexC**												
SSeneg12678	B	Tetra	46	29	121–377	0.37	0.96	0.95	0.02	0.44	*	Yes
SSeneg11209	G	Tetra	54	10	94–134	0.78	0.79	0.76	0.20	0.01	(*)	ns
SSeneg433	Y	Tetra	54	6	101–174	0.56	0.62	0.57	0.44	0.05	(*)	ns
SSeneg7919	Y	Tetra	53	9	174–210	0.53	0.79	0.75	0.23	0.2	(*)	Yes
SSeneg1973	Y	Tetra	53	23	249–329	0.94	0.93	0.92	0.04	− 0.01	(*)	ns
SSeneg17673	R	Tetra	54	5	116–177	0.44	0.73	0.67	0.35	0.24	*	Yes
SSeneg10308	R	Tetra	54	13	161–239	0.87	0.90	0.88	0.08	0.01	ns	ns
**MultiplexD**												
SSeneg1505	B	Tetra	57	7	112–136	0.75	0.78	0.75	0.23	0.01	ns	ns
SSeneg4306	B	Tetra	57	2	204–208	0.51	0.50	0.37	0.72	− 0.01	ns	ns
SSeneg10667	B	Tetra	54	7	277–301	0.56	0.64	0.61	0.36	0.06	ns	ns
SSeneg2307	G	Tetra	57	6	134–166	0.53	0.55	0.50	0.51	0.02	(ns)	ns
SSeneg13116	G	Tetra	57	7	199–235	0.63	0.72	0.69	0.28	0.06	ns	ns
SSeneg1201	Y	Penta	57	13	115–180	0.40	0.83	0.81	0.16	0.35	*	Yes
SSeneg4572	Y	Tetra	57	2	207–215	0.26	0.48	0.36	0.73	0.29	*	Yes
SSeneg4065	R	Tetra	57	10	117–161	0.86	0.84	0.82	0.14	− 0.01	ns	ns
SSeneg8782	R	Tetra	57	10	200–242	0.88	0.83	0.81	0.15	− 0.03	ns	ns
**MultiplexE**												
SSeneg5850	B	Tetra	50	4	74–92	0.62	0.62	0.55	0.49	0	ns	ns
SSeneg2473	B	Tetra	50	4	204–216	0.70	0.56	0.46	0.61	− 0.12	ns	ns
SSeneg544	B	Tetra	50	4	282–290	0.70	0.61	0.53	0.52	− 0.08	ns	ns
SSeneg87	G	Tetra	50	12	106–166	0.64	0.67	0.62	0.37	0.02	ns	ns
SSeneg5828	G	Tetra	49	7	192–224	0.55	0.48	0.46	0.52	− 0.11	ns	ns
SSeneg3415	Y	Tetra	50	8	94–132	0.56	0.64	0.60	0.39	0.06	(ns)	ns
SSeneg5919	Y	Di	50	8	204–224	0.66	0.74	0.69	0.31	0.05	ns	ns
SSeneg585	R	Tetra	50	7	103–127	0.62	0.75	0.71	0.27	0.07	(*)	Yes
SSeneg14542	R	Tetra	49	8	202–244	0.67	0.76	0.71	0.29	0.06	ns	ns
**MultiplexF**												
SSeneg1411	B	Tetra	64	3	120–128	0.22	0.25	0.23	0.78	0.05	ns	ns
SSeneg3069	B	Tetra	63	13	183–245	0.73	0.87	0.85	0.11	0.08	(*)	ns
SSeneg9009	B	Tetra	64	19	286–368	0.89	0.93	0.92	0.04	0.02	ns	ns
SSeneg437	G	Tetra	65	9	219–249	0.52	0.81	0.78	0.19	0.22	ns	Yes
SSeneg247	Y	Tetra	61	7	85–122	0.71	0.72	0.68	0.30	− 0.02	ns	ns
SSeneg73	Y	Di	65	16	199–255	0.85	0.86	0.84	0.12	0.01	ns	ns
SSeneg12624	Y	Penta	64	11	311–359	0.84	0.82	0.79	0.17	− 0.02	ns	ns
SSeneg12095	R	Tetra	65	4	148–160	0.51	0.48	0.42	0.61	− 0.03	ns	ns
SSeneg582	R	Di	62	18	224–308	0.94	0.87	0.85	0.11	− 0.05	ns	ns
**MultiplexG**												
SSeneg3683	B	Tetra	69	11	125–167	0.75	0.82	0.80	0.15	0.04	ns	ns
SSeneg5713	B	Di	65	21	227–311	0.86	0.88	0.87	0.08	0.01	ns	ns
SSeneg1667	G	Di	69	24	225–319	0.80	0.89	0.88	0.07	0.05	(ns)	Yes
SSeneg2891	Y	Tetra	68	9	150–190	0.65	0.82	0.79	0.19	0.11	*	Yes
SSeneg45	Y	Tetra	69	5	242–258	0.59	0.65	0.59	0.44	0.04	(*)	ns
SSeneg12417	R	Di	69	9	199–225	0.86	0.78	0.74	0.25	− 0.06	(ns)	ns
SSeneg10524	R	Tetra	69	7	266–286	0.75	0.71	0.66	0.34	− 0.05	ns	ns
**MultiplexH**												
SSeneg4608	B	Tetra	71	4	82–104	0.13	0.16	0.15	0.86	0.14	ns	ns
SSeneg2868	B	Tetra	71	9	112–172	0.78	0.83	0.80	0.17	0.03	(*)	ns
SSeneg11316	B	Tetra	71	10	214–292	0.72	0.71	0.67	0.33	0.00	(*)	ns
SSeneg287	G	Tetra	71	7	68–114	0.55	0.52	0.47	0.55	− 0.05	*	ns
SSeneg90	G	Tetra	71	13	133–175	0.93	0.85	0.84	0.13	− 0.05	ns	ns
SSeneg2596	Y	Tetra	71	5	78–104	0.38	0.40	0.35	0.68	0.00	*	ns
SSeneg8412	Y	Tetra	71	8	138–172	0.41	0.47	0.43	0.57	0.05	(*)	ns
SSeneg6827	R	Tetra	71	4	88–100	0.32	0.33	0.30	0.71	− 0.02	ns	ns
SSeneg5412	R	Tetra	71	7	148–216	0.41	0.46	0.43	0.57	0.05	*	ns
**MultiplexI**												
SSeneg854	B	Di	69	6	85–95	0.52	0.60	0.53	0.50	0.07	ns	ns
SSeneg5899	B	Tetra	69	5	164–216	0.41	0.47	0.43	0.58	0.06	ns	ns
SSeneg5346	B	Di	68	43	184–542	0.87	0.95	0.94	0.02	0.04	(*)	ns
SSeneg1669	G	Tetra	69	16	94–168	0.75	0.82	0.80	0.15	0.05	ns	ns
SSeneg7074	G	Tetra	69	6	144–182	0.64	0.76	0.71	0.30	0.08	ns	Yes
SSeneg4382	Y	Tetra	64	5	92–108	0.22	0.42	0.37	0.65	0.31	*	Yes
SSeneg53551	Y	Tetra	67	8	142–184	0.72	0.72	0.68	0.30	0.00	ns	ns
SSeneg3978	R	Tetra	69	7	84–108	0.67	0.68	0.64	0.34	0.00	ns	ns
SSeneg15332	R	Tetra	68	19	168–250	0.91	0.89	0.87	0.08	− 0.02	ns	ns
**MultiplexJ**												
SSeneg17159	B	Tetra	58	5	75–93	0.48	0.47	0.42	0.59	− 0.03	ns	ns
SSeneg9042	B	Tetra	56	19	174–260	0.77	0.86	0.83	0.12	0.06	ns	ns
SSeneg1723	G	Tetra	58	7	97–127	0.55	0.51	0.47	0.53	− 0.08	ns	ns
SSeneg348796	Y	Tetra	58	6	81–101	0.78	0.67	0.61	0.41	− 0.09	(ns)	ns
SSeneg7987	Y	Di	58	32	238–354	0.88	0.94	0.93	0.03	0.03	(*)	Yes
SSeneg3077	R	Tetra	58	4	94–110	0.40	0.36	0.33	0.68	− 0.07	(ns)	ns
SSeneg10804	R	Tetra	54	23	261–525	0.93	0.87	0.85	0.10	− 0.04	ns	ns
**MultiplexK**												
SSeneg2083	B	Tetra	62	9	92–124	0.69	0.65	0.61	0.36	− 0.05	ns	ns
SSeneg4083	B	Tetra	63	6	220–242	0.56	0.58	0.54	0.43	0.00	(ns)	ns
SSeneg171	G	Tetra	63	7	136–172	0.78	0.75	0.71	0.28	− 0.03	ns	ns
SSeneg2487	G	Tetra	50	26	188–328	0.98	0.95	0.93	0.03	− 0.02	*	ns
SSeneg566	Y	Tetra	63	7	114–136	0.84	0.77	0.73	0.27	− 0.05	ns	ns
SSeneg6876	R	Tetra	63	21	108–198	0.94	0.91	0.90	0.06	− 0.02	ns	ns
SSeneg4081	R	Tetra	61	19	268–374	0.90	0.88	0.87	0.08	− 0.02	ns	ns
**MultiplexL**												
SSeneg7666	B	Di	46	21	162–224	0.89	0.92	0.90	0.05	0.01	ns	ns
SSeneg4003	B	Di	46	21	244–332	0.89	0.93	0.91	0.05	0.01	ns	ns
SSeneg5891	G	Tetra	46	12	97–159	0.76	0.78	0.75	0.22	0.01	(ns)	ns
SSeneg774	G	Tetra	46	4	172–178	0.17	0.27	0.26	0.75	0.26	*	Yes
SSeneg6689	Y	Tetra	44	5	111–131	0.11	0.41	0.38	0.61	0.55	(*)	Yes
SSeneg1147	Y	Tetra	46	14	204–252	0.80	0.91	0.89	0.06	0.06	ns	Yes
SSeneg14333	R	Tetra	46	8	132–172	0.37	0.83	0.79	0.18	0.38	*	Yes
SSeneg2996	R	Tetra	45	14	229–291	0.64	0.90	0.88	0.07	0.16	(*)	Yes
**MultiplexM**												
SSeneg506	B	Tetra	63	6	88–114	0.22	0.66	0.60	0.43	0.49	*	Yes
SSeneg387243	B	Tetra	62	17	250–316	0.86	0.87	0.85	0.11	0.01	ns	ns
SSeneg10877	G	Tetra	63	12	177–223	0.71	0.80	0.77	0.19	0.04	*	ns
SSeneg14597	G	Tetra	62	13	250–356	0.76	0.90	0.89	0.07	0.08	*	Yes
SSeneg4328	Y	Tetra	63	16	96–168	0.92	0.92	0.90	0.06	− 0.01	(ns)	ns
SSeneg4039	Y	Di	60	26	248–322	0.43	0.91	0.90	0.05	0.36	*	Yes
SSeneg1988	R	Tetra	62	2	91–95	0.02	0.02	0.02	0.98	0.00	ns	na

Fluorescent labelling (B, blue; G, green; Y, yellow; R, red), repeat motif (Di, tetra or pentanucleoide), Number of samples (N), number of alleles (k), Allelic range, observed heterozygosity (Ho) and expected heterozygosity (He), polymorphic information content (PIC), non-exclusion probability of pair parent (NE-PP); null allele frequency (F(N)). Hardy–Weinberg equilibrium (HW; *significant after bonferroni correction; ns, non-significant) and Null alleles as determined by micro-checker (yes, significant after bonferroni correction; ns, non-significant).

To identify the genes close to the SSRs, the contigs selected for primer design were compared with Senegalese sole transcriptome and *C. semilaevis* genome. The analysis indicated a high degree of gene synteny conservation (higher than 90% in most multiplex PCRs) between *S. senegalensis* transcripts and *C. semilaevis* genes (Supplementary Table S4). Some of genes identified are of interest for aquaculture due to their role the role in immune response (toll-like receptor 3, interleukin-27 subunit beta, chemokine-like receptor 1, C-type mannose receptor 2 isoform X1), hormonal signalling (thyroid hormone receptor alpha-B, retinoic acid receptor RXR-alpha, retinol dehydrogenase 10, retinol dehydrogenase 8), antioxidant defences (superoxide dismutase [Cu–Zn]) or larval survival (high choriolytic enzyme 1), epigenetics (betaine–homocysteine S-methyltransferase 1), reproduction (Prostaglandin E synthase 3) or sensing (taste receptor type 1 member 1).

### Design of supermultiplex for parentage assignment

**Figure 2:**
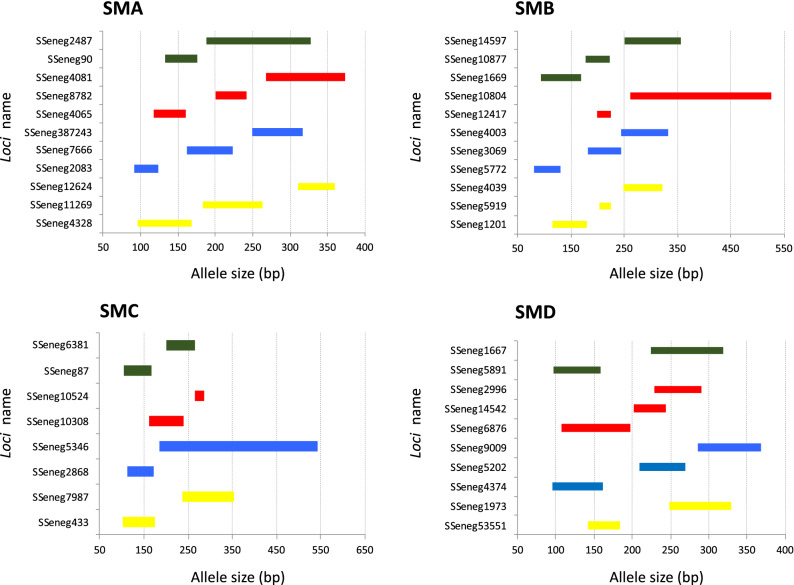
Allelic ranges of the 40 SSRs selected for the supermultiplex (SM) PCRs. The markers are shown by SM (**A**–**D**).

To design high variable PCR multiplex assays (named as supermultiplex) suitable for pedigree reconstruction in breeding programs, a subset of 40 out of 106 markers was selected according to their allelic range and genetic variability markers and they were rearranged in four supermultiplex assays (referred from SMA, SMB, SMC and SMD) ranging from 8- to 11-plex. Allelic allelic ranges are depicted in Fig. 2. Genetic characteristics are shown in Supplementary Table S5. As average, PIC information in the four supermultiplex ranged between 0.79–0.82 and 73% of markers had a PIC value higher than 0.8 and 89% higher than 0.7 (Supplementary Table S5). In total, motifs of 9 markers were dinucleotide, 29 tetranucleotide and 2 pentanucleotide. According to the synteny analysis these markers were positioned in 17 out of 21 chromosomes.

**Figure 3:**
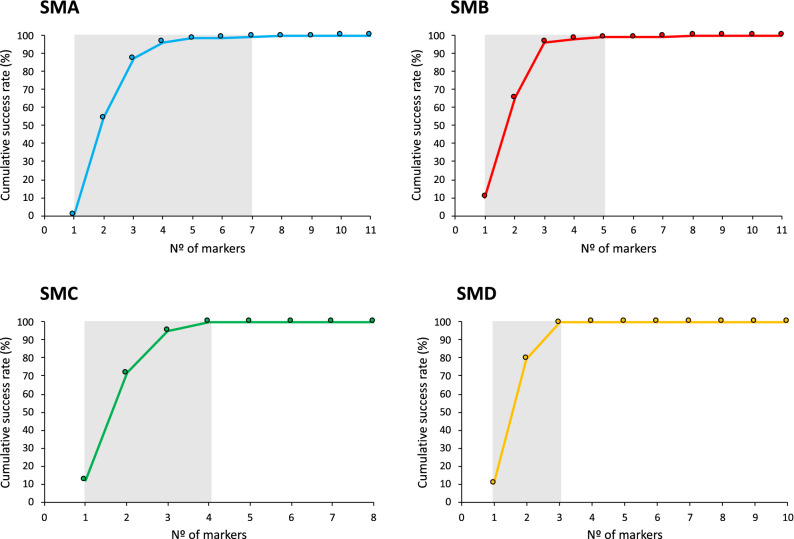
Cumulative success rate for parentage assignment based on exclusion with markers ranked on PIC value. The grey area indicates the *loci* required to reach more than 99% probability of assigning a correct parent–offspring relationship. SMA n = 92 parents; SMB, n = 15 parents; SMC, n = 15; SMD, n = 15.

In order to validate the usefulness of the four supermultiplex for parentage assignment in sole, they were tested using different set of parents and offspring. In the case of SMA, an offspring set of 100 individuals and 92 putative parents from 4 different broodstocks (48 females and 44 males) were 100% assigned using to a single parent pair without observing null allele mismatches. For SMB, SMC and SMD, a broodstock tank of 15 parents was characterized and 5 offspring were 100% assigned to a single pair without mismatches. Ranking markers using PIC resulted in accumulative success rate higher than 99% with 7, 5, 4 and 3 markers in SMA, SMB, SMC and SMD, respectively (Fig. 3).

### Construction of an integrated genetic map and synteny analysis

**Figure 4:**
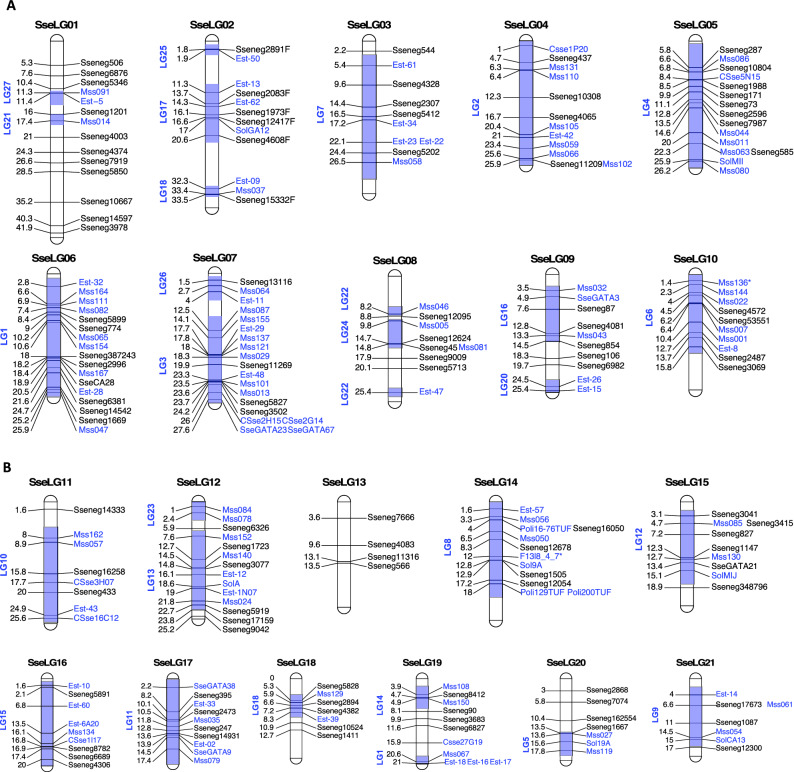
Integrated SSR genetic map of Senegalese sole (*S. senegalensis*). SseLG refer to the linkage groups according the high-density SNP genetic map. Genetic distance is indicated on the left. SSRs of this study are indicate in black and those from Molina-Luzón et al., 2015 in blue. The LGs previously assigned to these markers are shaded and indicated on the left. (**a**) SseLG1- SseLG10; (**b**) SseLG11- SseLG21.

To construct the integrated genetic map, 121 out of 129 SSRs reported by Molina-Luzon, et al.^14^ were succesfully mapped onto the LR-hybrid female genome (Supplementary Table S2 tab "PrimerMappingLuzon"). Overall, a total of 229 SSRs (108 of this study + 121 previously published) were located in genome scaffolds anchored to the 21 linkage groups (SseLGs) of a recenlty high-density SNP genetic linkage map built in the lab that matches with the expected number of chromosomes *S. senegalensis*. The number of markers per LG ranged from 4 located in SseLG13 to 19 in SseLG07 (Table 3; Fig. 4a,b; Supplementary Table S2 tab "Physical_genetic_map"). Eight markers were located in unplaced scaffolds. Interestingly, marker distribution in the SseLGs was highly conincident with LGs of Molina-Luzon, et al.^14^. Only those markers from LG1 were split into the SseLG6 and SseLG19 probably due to a misarrangement in the previous map since these markers moved as two blocks between SseLGs.

**Table 3:** SSR distribution.

High density SNP map	SSR markers			*Cynoglossus* Chromosomes	LD genetic map
	This study	LD genetic map	Total		
SseLG01	11	3	14	chr3,chr20	LG21,LG27
SseLG02	6	6	12	chr14,chr16	LG17,LG18,LG25
SseLG03	5	5	10	chr1, chr8, chrZ	LG7
SseLG04	4	8	12	chr11, chrZ	LG2
SseLG05	8	7	15	chrZ	LG4
SseLG06	7	10	17	chr9	LG1
SseLG07	4	15	19	chr5	LG3,LG26
SseLG08	5	4	9	chr4	LG22,LG24
SseLG09	5	5	10	chr13	LG16,LG20
SseLG10	4	6	10	chr6	LG6
SseLG11	3	5	8	chr10	LG10
SseLG12	6	8	14	chr15	LG13,LG23
SseLG13	4	0	4	chr19	–
SseLG14	4	8	12	chr2	LG8
SseLG15	5	4	9	chr12	LG12
SseLG16	4	5	9	chr1	LG15
SseLG17	4	6	10	chr7	LG11
SseLG18	5	2	7	chr8	LG19
SseLG19	4	7	11	chr17	LG1,LG14
SseLG20	4	3	7	chr18	LG5
SseLG21	3	4	7	chr14	LG9
Unplaced	3	5	8	–	
Total	108	126	234		

Markers are groups by the 21 linkage groups (SseLG) of the high-density SNP genetic map. The number of SSRs of this study and those from Low-density (LD) genetic linkage map (Molina-Luzon et al., 2015) are indicated. The location of markers in *C. semilaevis* genome by blasting the scaffold containing the SSR marker and the LG in the LD genetic map are indicated.

Macrosynteny analysis bewteen *S. senegalensis* and *C. semilaevis* chromosomes demonstrated that 17 SseLGs of *S. senegalensis* matched perfectly with different chromosomes of *C. semilaevis* (Table 3). Only four chromosomes in *S. senegalensis* appeared as chromosomal rearrangements of C*. semilaevis* and the sequences of Z chromosome were dispersed through the SseLG3, SseLG4 and SseLG5. The SseLG1 appeared as a fusion of chromosomes 3 and 20 of *C. semilaevis*. Moreover, some rearrangements were observed for SseLG2 that included the chromosome 16 and part of 14, the SseLG3 that grouped regions of chromosomes 1, 8 and Z and the SseLG4 that combined the chromosome 11 and regions of Z.

## Discussion

The SSRs are highly abundant in the genome of vertebrates although their use has been limited by the knowledge of flanking regions suitable for primer design. Some authors considered as alternative the cross-species amplification of highly conserved SSRs^14,46,47^. Recently, a study in Senegalese sole based on the 1.1% of the genome information estimated a high density of SSRs (675 per Mb) with dinucleotide SSRs representing overall 59.7%^15^. In this study, we took advantage of a 85 k genome draft^7^ and a de novo female hybrid genome based on Nanopore and Illumina reads to overpass the deficit of markers in Senegalese sole. Total size of this new genome was 608 Mb very close to the 600.3 Mb reported for the 85 k Illumina assembly^7^ suggesting that Senegalese sole genome is a slightly bigger than other flatfish (up to 584 Mb)^2–4,48^ . This assembly had a high-quality gene representativity (completeness was 96.2% similar to previous flatfish assemblies)^3^ with the marker density of 886.7 SSRs per megabase (Supplementary Table S1 tab "SSR_genome"). Previous cytogenetic analyses demonstrated that most of di- and tetranucleotides appear widely distributed in subtelomeric position of metacentric, submetacentric and acrocentric chromosomes^15^ and hence both of them were considered suitable for primer design and multiplex amplification in this study.

Whole-genome mapping requires high-throughput strategies to save consumables, labour costs and reduce the processing and analysis times. PCR multiplex assays have been successfully developed in seabream^25,49^ and grapevine^20^ for QTLs identification and pedigree reconstruction. In this study, thirteen PCR multiplex assays comprising 106 markers widespread in the genome were optimized. Although previous studies in sole have reported microsatellite markers derived from EST or SSR-enriched libraries^46,50,51^ only three of them considered SSR multiplexing (from 4 to 8-pex)^47,52,53^. These new multiplex PCRs and their integration with the 121 markers previously published^14^ represent key genomic tools for QTL detection in sole. The new genome information provided also facilitates the integration with SNP markers and the redesign of some SSR primers in the map to construct new multiplexes that improve the genome coverage.

Tetra- and pentanucleotides predicted motifs were initially selected for multiplex PCRs although finally some of them (12%) followed a dinucleotide allelic series. It has been demonstrated that SSRs with dinucleotide motifs have a higher variability but more prone to genotyping errors than those with larger motifs^28,54^. In this study, the average number of alleles per locus was 10.9 ranging from 2 to 43 in accordance with previous SSR markers in Senegalese sole^14,46,50,51^. As expected, the dinucleotide markers showed a higher variability (average PIC 0.84) than tetra- (0.65) and pentanucleotides (0.66). Moreover, scoring accuracy was estimated using a standardized methodology to identify potential errors in the electropherograms^21^ indicating only a small set of markers (17) with stuttering, allele dropout or intermediate alleles, ~ 16% of total markers. In seabream, the percentage of *loci* with some of these errors was similar although with higher rates of intermediate alleles^21^. It should be indicated that stutter peaks have a low effect to assign *loci* size in tetranucleotides as observed by a double validation across two independent labs reaching similar values in genetic diversity parameters.

The use of genetic tools to infer genealogies is a demand for genetic breeding programs in mass-spawning species such as Senegalese sole. Due to the economic value of these species, the optimization of genotyping tools for parental assignment in a feasible, accurate and cost-effective way is a requirement. Moreover, the loss in variability that occurs in subsequent selection cycles makes necessary a minimal number of markers to sustain the program through some generations. Both the number of *loci* and their heterozygosity level may influence the power of markers for parentage exclusion approaches^55^. In this study, a total of 40 high variable and genome widespread markers were selected according to PIC and combined in four supermultiplex (7 to 11-pex). Assignment simulations indicated that a subset of 7, 5, 4 and 3 markers were able to assign 99% offspring with SMA (11-pex), SMB (11-pex), SMC (8-pex) or SMD (10-pex), respectively. Moreover, a real testing using SMA to genotype 92 parents accurately allocated all 100 parent–offspring relationships. All these data indicate that these supermultiplex can be transferred to the industry as standards for pedigree reconstruction to support a long-term use for genetic breeding selection.

An integrated genetic map with 229 SSR markers was generated that improve the current low density genetic linkage map available in Senegalese sole^14^ (Fig. 4). Using a high-density SNP genetic map as reference, the whole set of SSR markers was distributed in 21 LGs that fit with the haploid complement in this flatfish species (3 metacentric pairs, 2 submetacentric pairs, 4 subtelocentric pairs and 12 acrocentric pairs)^56^. Our anaysis confirmed that the LGs from the previous genetic map^14^ clustered perfectly within the SseLGs after anchoring the LR-hybrid female genome and the high density genetic map (Fig. 4 and Table 3). Only LG1 was split into two SseLGs that might be due to an error in the consensus between gynogenetic families.

Flatfish genome comparisons have demonstrated a high degree of conservation at macrosynteny level^5,57,58^. Our data confirmed that most of chromosomes matched one-by-one with different chromosomes of *C. semilaevis* supporting this high conservation observed in other flatfish. Moreover, chromosome fusions and translocations have occured frequently during flatfish evolution shaping the number of chromosomes from n = 24 pairs in Japanese flounder to n = 20 autosome pairs and one sexual chromosome pair in C*. semilaevis*. In *S. senegalensis*, it has been hypothesized that the largest metacentric chromosome arose from a robertsonian fusion of two acrocentric chromosomes followed by pericentric inversions^16,59^. Our data also support this fusion and chromosome rearrangements between chromosomes 3 and 20 of *C. semilaevis* (Table 3). It should be noted that Senegalese sole has two additional metacentric pairs and 2 submetacentric pairs unlike *C. semilaevis* with all chromosomes telocentric^60^. Three LGs (SseLG02, SseLG03 and SseLG04) were also associated with more than one chromosome of *C. semilaevis* and a fourth LG (SseLG05) was syntenic with the large sexual chromosome Z (Table 3). Some robertsonian translocations (fissions and fusions) could be the origin of these non-acrocentric chromosomes in *S. senegalensis* as previously observed in turbot^5^. Most interestingly, the high remodelling of sexual ZW chromosomes that was also previously assessed by a scaffold mapping strategy^8^ suggests that a shift in the sex determining system might have occurred in Senegalese sole. In fact, a sex determination XX-XY system was proposed in this species with the female as homogametic sex^8,61^. Although the SseLG01 has been proposed as a sex proto-chromosome due to the location of some key sex-determining genes and repetitive sequences^12,16^, the spreading of Z/W sequences through the genome indicates that a further experimental validation is required to identify a putative major *loci* for sex determination.

In conclusion, this study uses two genome assemblies of Senegalese sole for the identification of SSR markers, sequence validation and cross-species synteny comparison analysis. A total of 106 selected SSR markers were structured in thirteen multiplex PCR assays available for whole-genome mapping. Moreover, forty high-polymorphic markers were used to optimize four high-variable supermultiplex PCRs suitable for pedigree analysis and genetic breeding programs. All SSR markers were positioned in the genome and integrated with previous published SSR markers to generate a new integrated genetic map containing 21 LGs. A macrosynteny comparison with *C. semilaevis* indicated the largest metacentric and submetacentric chromosomes of *S. senegalensis* could be explained by fusions and rearrangements of telocentric chromosomes in C. semilaevis. This integrated genetic map and the new multiplex PCRs provide a valuable resource for association studies, selection breeding and flatfish comparative genomics.

## Supplementary Information

Supplementary Information 1.

Supplementary Information 2.

Supplementary Information 3.

Supplementary Information 4.

Supplementary Information 5.

Supplementary Information 6.

Supplementary Information 7.

## References

[1] Cerda J, Manchado M (2013). Advances in genomics for flatfish aquaculture. Genes Nutr..

[2] Chen S (2014). Whole-genome sequence of a flatfish provides insights into ZW sex chromosome evolution and adaptation to a benthic lifestyle. Nat. Genet..

[3] Xu XW (2020). Draft genomes of female and male turbot *Scophthalmus maximus*. Sci. Data.

[4] Shao C (2017). The genome and transcriptome of Japanese flounder provide insights into flatfish asymmetry. Nat. Genet..

[5] Maroso F (2018). Highly dense linkage maps from 31 full-sibling families of turbot (*Scophthalmus maximus*) provide insights into recombination patterns and chromosome rearrangements throughout a newly refined genome assembly. DNA Res..

[6] Robledo D, Rubiolo JA, Cabaleiro S, Martinez P, Bouza C (2017). Differential gene expression and SNP association between fast- and slow-growing turbot (*Scophthalmus maximus*). Sci. Rep..

[7] Manchado M, Planas JV, Cousin X, Rebordinos L, Claros MG, Mackenzie S, Jentoft S (2016). Genomics in Aquaculture.

[8] Manchado M, Planas JV, Cousin X, Rebordinos L, Claros MG, Muñoz-Cueto J, Mañanós-Sánchez E, Sánchez-Vázquez F (2019). The Biology of Sole.

[9] Souissi A, Bonhomme F, Manchado M, Bahri-Sfar L, Gagnaire PA (2018). Genomic and geographic footprints of differential introgression between two divergent fish species (*Solea* spp.). Heredity.

[10] Roman-Padilla J, Rodriguez-Rua A, Claros MG, Hachero-Cruzado I, Manchado M (2016). Genomic characterization and expression analysis of four apolipoprotein A-IV paralogs in Senegalese sole (*Solea senegalensis* Kaup). Comp. Biochem. Physiol. B Biochem. Mol. Biol..

[11] Carballo C (2019). Genomic and phylogenetic analysis of choriolysins, and biological activity of hatching liquid in the flatfish Senegalese sole. PLoS ONE.

[12] Portela-Bens S (2017). Integrated gene mapping and synteny studies give insights into the evolution of a sex proto-chromosome in *Solea senegalensis*. Chromosoma.

[13] Cordoba JM, Chavarro C, Schlueter JA, Jackson SA, Blair MW (2010). Integration of physical and genetic maps of common bean through BAC-derived microsatellite markers. BMC Genom..

[14] Molina-Luzon MJ (2015). First haploid genetic map based on microsatellite markers in Senegalese sole (*Solea senegalensis*, Kaup 1858). Mar. Biotechnol. (NY).

[15] Garcia E (2019). Integrative genetic map of repetitive DNA in the sole *Solea senegalensis* genome shows a Rex transposon located in a proto-sex chromosome. Sci. Rep..

[16] Rodriguez ME (2019). Evolution of the proto sex-chromosome in *Solea senegalensis*. Int. J. Mol. Sci..

[17] Wang W (2015). High-density genetic linkage mapping in turbot (*Scophthalmus maximus* L.) based on SNP markers and major sex- and growth-related regions detection. PLoS ONE.

[18] Lu Q (2019). Genome-wide identification of microsatellite markers from cultivated peanut (*Arachis hypogaea* L.). BMC Genom..

[19] Sundaray JK (2016). Simple sequence repeats (SSRs) markers in fish genomic research and their acceleration via next-generation sequencing and computational approaches. Aquac. Int..

[20] Zarouri B (2015). Whole-genome genotyping of grape using a panel of microsatellite multiplex PCRs. Tree Genet. Genomes.

[21] Lee-Montero I (2013). Development of the first standardised panel of two new microsatellite multiplex PCRs for gilthead seabream (*Sparus aurata* L.). Anim. Genet..

[22] Carballo C (2020). Heritability estimates and genetic correlation for gowth traits and LCDV susceptibility in gilthead sea bream (*Sparus aurata*). Fishes.

[23] Garcia-Celdran M (2015). Estimates of heritabilities and genetic correlations of growth and external skeletal deformities at different ages in a reared gilthead sea bream (*Sparus aurata* L.) population sourced from three broodstocks along the Spanish coasts. Aquaculture.

[24] Lee-Montero I (2015). Genetic parameters and genotype-environment interactions for skeleton deformities and growth traits at different ages on gilthead seabream (*Sparus aurata* L.) in four Spanish regions. Anim. Genet..

[25] Negrin-Baez D, Negrin-Baez D, Rodriguez-Ramilo ST, Afonso JM, Zamorano MJ (2016). Identification of quantitative trait loci associated with the skeletal deformity LSK complex in gilthead seabream (*Sparus aurata* L.). Mar. Biotechnol..

[26] Benzekri H (2014). De novo assembly, characterization and functional annotation of Senegalese sole (*Solea senegalensis*) and common sole (*Solea solea*) transcriptomes: Integration in a database and design of a microarray. BMC Genom..

[27] Flores-Renteria L, Krohn A (2013). Scoring microsatellite loci. Methods Mol. Biol..

[28] Nater A, Kopps AM, Krutzen M (2009). New polymorphic tetranucleotide microsatellites improve scoring accuracy in the bottlenose dolphin *Tursiops aduncus*. Mol. Ecol. Resour..

[29] Wood DE, Salzberg SL (2014). Kraken: Ultrafast metagenomic sequence classification using exact alignments. Genome Biol..

[30] Marco-Sola S, Sammeth M, Guigo R, Ribeca P (2012). The GEM mapper: Fast, accurate and versatile alignment by filtration. Nat. Methods.

[31] Vaser R, Sovic I, Nagarajan N, Sikic M (2017). Fast and accurate de novo genome assembly from long uncorrected reads. Genome Res..

[32] Li H (2018). Minimap2: pairwise alignment for nucleotide sequences. Bioinformatics.

[33] Zimin AV (2017). Hybrid assembly of the large and highly repetitive genome of *Aegilops tauschii*, a progenitor of bread wheat, with the MaSuRCA mega-reads algorithm. Genome Res..

[34] Zimin AV (2013). The MaSuRCA genome assembler. Bioinformatics.

[35] Miller JR (2008). Aggressive assembly of pyrosequencing reads with mates. Bioinformatics.

[36] Simao FA, Waterhouse RM, Ioannidis P, Kriventseva EV, Zdobnov EM (2015). BUSCO: Assessing genome assembly and annotation completeness with single-copy orthologs. Bioinformatics.

[37] Waterhouse RM (2018). BUSCO applications from quality assessments to gene prediction and phylogenomics. Mol. Biol. Evol..

[38] Claros MG, Seoane P, Manchado M (2020). figshare.

[39] Beier S, Thiel T, Munch T, Scholz U, Mascher M (2017). MISA-web: a web server for microsatellite prediction. Bioinformatics.

[40] Sanchez JJ (2003). Multiplex PCR and minisequencing of SNPs–a model with 35 Y chromosome SNPs. Forensic. Sci. Int..

[41] Kalinowski ST, Taper ML, Marshall TC (2007). Revising how the computer program CERVUS accommodates genotyping error increases success in paternity assignment. Mol. Ecol..

[42] Peakall R, Smouse PE (2012). GenAlEx 6.5: genetic analysis in excel. Population genetic software for teaching and research—an update. Bioinformatics.

[43] Van Oosterhout C, Hutchinson WF, Wills DPM, Shipley P (2004). Micro-checker: Software for identifying and correcting genotyping errors in microsatellite data. Mol. Ecol. Notes.

[44] Sekino M, Kakehi S (2012). PARFEX v1.0: An EXCEL-based software package for parentage allocation. Conserv. Genet. Resour..

[45] Ouellette LA, Reid RW, Blanchard SG, Brouwer CR (2018). LinkageMapView-rendering high-resolution linkage and QTL maps. Bioinformatics.

[46] Funes V, Zuasti E, Catanese G, Infante C, Manchado M (2004). Isolation and characterization of ten microsatellite loci for Senegal sole (*Solea senegalensis* Kaup). Mol. Ecol. Notes.

[47] Castro J (2006). A microsatellite marker tool for parentage analysis in Senegal sole (*Solea senegalensis*): Genotyping errors, null alleles and conformance to theoretical assumptions. Aquaculture.

[48] Figueras A (2016). Whole genome sequencing of turbot (*Scophthalmus maximus*; Pleuronectiformes): A fish adapted to demersal life. DNA Res..

[49] Negrin-Baez D (2015). A set of 13 multiplex PCRs of specific microsatellite markers as a tool for QTL detection in gilthead seabream (*Sparus aurata* L.). Aquac Res..

[50] Molina-Luzon MJ (2012). Validation and comparison of microsatellite markers derived from Senegalese sole (*Solea senegalensis*, Kaup) genomic and expressed sequence tags libraries. Mol. Ecol. Resour..

[51] Chen S-L, Shao C-W, Xu G-B, Liao X-L, Tian Y-S (2008). Development of 15 novel dinucleotide microsatellite markers in the Senegalese sole *Solea senegalensis*. Fish. Sci..

[52] Porta J, Porta JM, Martínez-Rodríguez G, Álvarez MC (2006). Development of a microsatellite multiplex PCR for Senegalese sole (*Solea senegalensis*) and its application to broodstock management. Aquaculture.

[53] De La Herran R (2008). A highly accurate, single PCR reaction for parentage assignment in Senegal sole based on eight informative microsatellite loci. Aquacult. Res..

[54] Zalapa JE (2012). Using next-generation sequencing approaches to isolate simple sequence repeat (SSR) loci in the plant sciences. Am. J. Bot..

[55] Labuschagne C, Nupen L, Kotze A, Grobler PJ, Dalton DL (2015). Assessment of microsatellite and SNP markers for parentage assignment in ex situ African Penguin (*Spheniscus demersus*) populations. Ecol. Evol..

[56] Merlo MA (2017). Analysis of the histone cluster in Senegalese sole (*Solea senegalensis*): Evidence for a divergent evolution of two canonical histone clusters. Genome.

[57] Bouza C (2012). An Expressed sequence Tag (EST)-enriched genetic map of turbot (*Scophthalmus maximus*): A useful framework for comparative genomics across model and farmed teleosts. BMC Genet..

[58] Garcia-Angulo A (2019). Genome and phylogenetic analysis of genes involved in the immune system of *Solea senegalensis*—Potential applications in aquaculture. Front. Genet..

[59] Garcia-Angulo A (2018). Evidence for a Robertsonian fusion in *Solea senegalensis* (Kaup, 1858) revealed by zoo-FISH and comparative genome analysis. BMC Genom..

[60] Zhang S, Wang C, Chu J (2004). C-banding pattern and nucleolar organizer regions of amphioxus *Branchiostoma belcheri* tsingtauense Tchang et Koo, 1936. Genetica.

[61] Viñas J, Asensio E, Piferrer F (2012). Gonadal sex differentiation in the Senegalese sole (*Solea senegalensis*) and first data on the experimental manipulation of its sex ratios. Aquaculture.

