# Supplementary material for: Development of whole-genome multiplex assays and construction of an integrated genetic map using SSR markers in Senegalese sole
Source: Sci Rep. 2020 Dec 14;10:21905. doi: 10.1038/s41598-020-78397-w (PMC7736592; doi:10.1038/s41598-020-78397-w)
Supplement: Supplementary file 1 — Supplementary Information 1. [file 41598_2020_78397_MOESM1_ESM.docx]

# Development of whole-genome multiplex assays and construction of an integrated genetic map using SSR markers in Senegalese sole

Israel Guerrero-Cózar^1^; Cathaysa Perez-Garcia^2^, Hicham Benzekri^3^, J. J. Sánchez^4^, Pedro Seoane^3^, Fernando Cruz^5^, Marta Gut^5^, Maria Jesus Zamorano^2^, M. Gonzalo Claros^3,6,7,8^, Manuel Manchado^1,9*^

^1^IFAPA Centro El Toruño, Junta de Andalucía, Camino Tiro Pichón s/n, 11500 El Puerto de Santa María, Cádiz, Spain

^2^Aquaculture Research Group (GIA), IU-ECOAQUA, Universidad de Las Palmas de Gran Canaria, Crta. Taliarte s/n, 35214 Telde, Spain

^3^Universidad de Málaga, Department of Molecular Biology and Biochemistry, Málaga, E-29071, Spain

^4^Instituto Nacional de Toxicología y Ciencias Forenses (INT), La Cuesta, La Laguna, Sta. Cruz de Tenerife, 38320, Spain

^5^CNAG-CRG, Centre for Genomic Regulation (CRG), Barcelona Institute of Science and Technology (BIST), Barcelona, 08028, Spain.

^6^CIBER de Enfermedades Raras (CIBERER), Málaga, E-29071, Spain

^7^Institute of Biomedical Research in Málaga (IBIMA), IBIMA-RARE, Málaga, E-29010, Spain

^8^Instituto de Hortofruticultura Subtropical y Mediterránea (IHSM-UMA-CSIC), Málaga, E-29010, Spain

^9^ “Crecimiento Azul", Centro IFAPA El Toruño, Unidad Asociada al CSIC”

*Corresponding author:

Manuel Manchado. IFAPA Centro *El Toruño*. Camino Tiro de Pichón s/n. 11500 El Puerto de Santa María (Cádiz), Spain. Tel: +34 671532088. Fax: +34 856102033. Email: [manuel.manchado@juntadeandalucia.es](mailto:carlos.infante@juntadeandalucia.es)^[[1]](#footnote-1)^

**Supplementary Table S1.** **SSR characterization in LR-hybrid female genome, preselected and selected contigs** (available as a separate MSExcel file).

SSR abundance and characteristics in the genome assembly and preselected contigs as determined by MISA are indicated (Tab SSR_genome). For preselected (Tab Preselected_contigs) and selected (Selected_contigs) contigs for SSR selection, the contig size, positioning with respect to *C. semilaevis* and number of SSR di-, tri-, tetra- and pentanucleotide repeats are indicated. Moreover, main figures about chromosome distribution, average contig distance and density of SSR markers (SSR/Mb) are presented. The parameters used for the analysis are indicated in each case. In "selected" SSR, synteny using the 85 kb genome and LR-hybrid female genome are indicated

**Supplementary Table S2. Primer information and mapping** (available as a separate MSExcel file).

Tabs "InitialMultiplexDesign" and "FinalMultiplex" contain the primer sequences, expected amplicon sizes, fluorescent labelling, contig names and positioning with respect to *C. semilaevis* in the initial and final design, respectively. The tab "Primer amounts" indicate the primer amounts and concentration of each *loci* in the multiplex PCRs. The tabs "PrimerMappingSSR" and "PrimerMappingLuzon" show the primer mapping in the female hybrid genome and the quality assessment for new SSRs designed in this study and those of Molina-Luzon et al., 2015, respectively. The tab "Physical_genetic_map" compares the position of each marker in the low-density genetic linkage map, *C. semilaevis* genome and high-density SNPs genetic linkage map.

**Supplementary Table S3. Genetic diversity estimates by multiplex PCRs (A-M) obtained by ULPGC** (available as a separate MSExcel file).

Allelic range, number of individuals analysed (N), number of alleles (k), observed (Ho) and expected (He), polymorphic information content (PIC), frequency of null alleles (F(N)) and Hardy-Weinberg (HW) equilibrium after bonferroni correction are indicated. ns, non-significant; * statistically significant.

**Supplementary Table S4. Gene linkage and synteny** (available as a separate MSExcel file).

For each multiplex PCR, the contigs name and the gene products found by blasting onto the *C. semilaevis* genome and Senegalese sole transcriptome are indicated.

**Supplementary Table S5. Genetic diversity estimates for supermultiplex PCRs (SMA-D) obtained by ULPGC** (available as a separate word file).

Polymorphic information content (PIC) and allelic range obtained by IFAPA and ULPGC laboratories, the repeat motif and chromosome location after synteny analysis with respect to *C. semilaevis* genome and positioning in the high-density SNPs genetic linkage map (SseLG) are indicated. Colours indicate the fluorescent labelling of each marker.

**Supplementary Fig S1. Electropherograms of thirteen multiplex PCRs (named from A to M)**. Loci names are indicated. (available as a separate pdf file).

1. [↑](#footnote-ref-1)
