# Supplementary material for: Development of whole-genome multiplex assays and construction of an integrated genetic map using SSR markers in Senegalese sole
Source: Sci Rep. 2020 Dec 14;10:21905. doi: 10.1038/s41598-020-78397-w (PMC7736592; doi:10.1038/s41598-020-78397-w)
Supplement: Supplementary file 2 — Supplementary Information 2. [file 41598_2020_78397_MOESM2_ESM.pdf]

Multiplex A

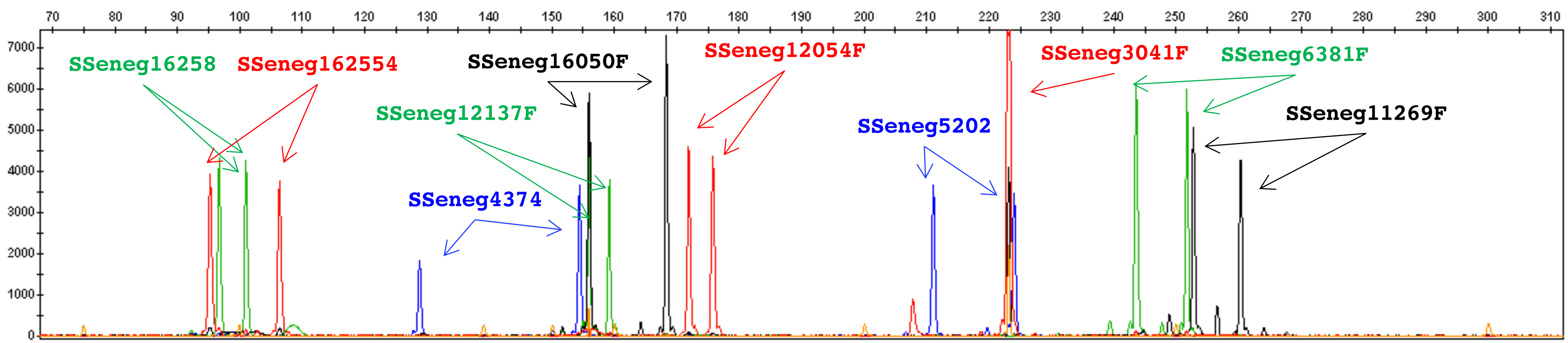

Multiplex B

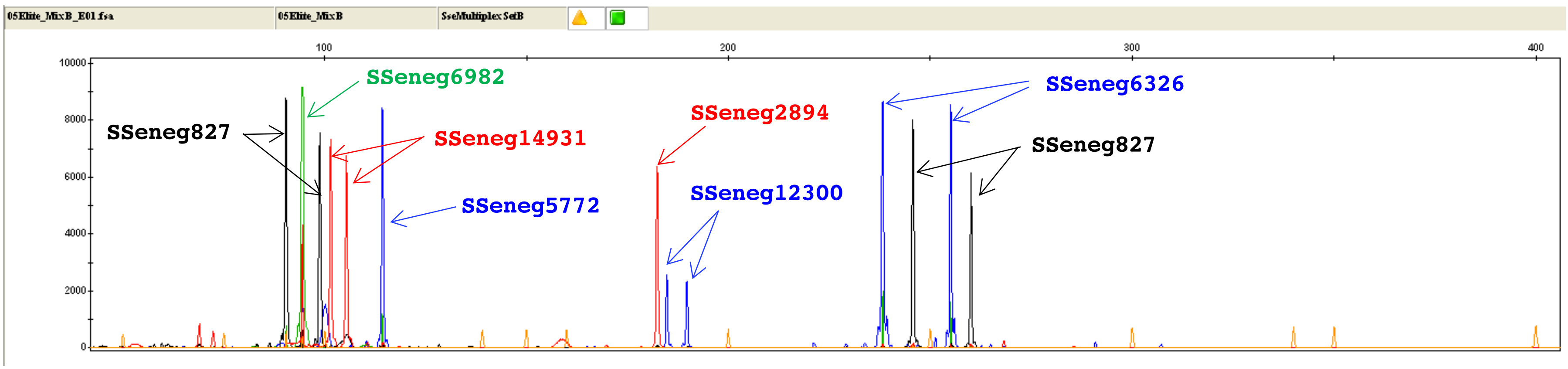

Multiplex C

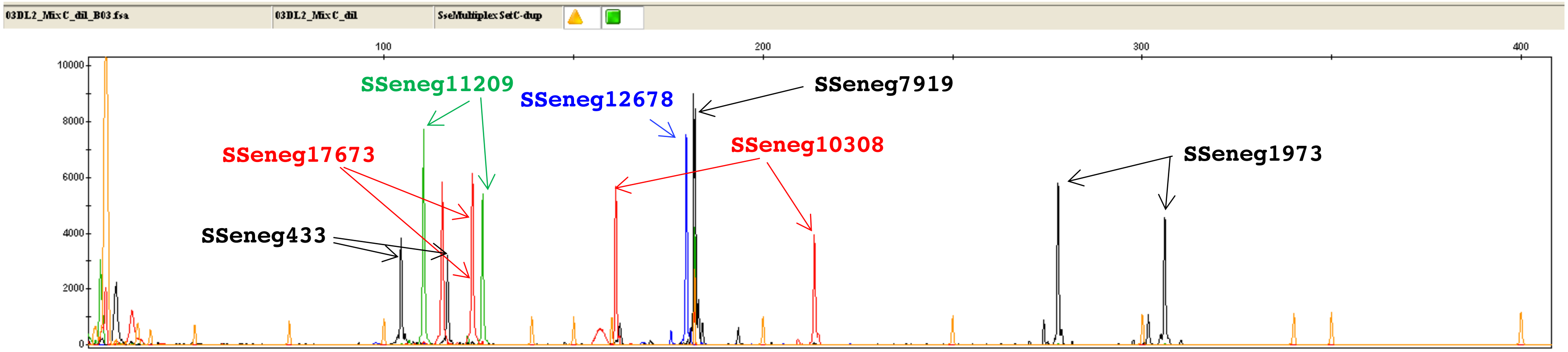

Multiplex D

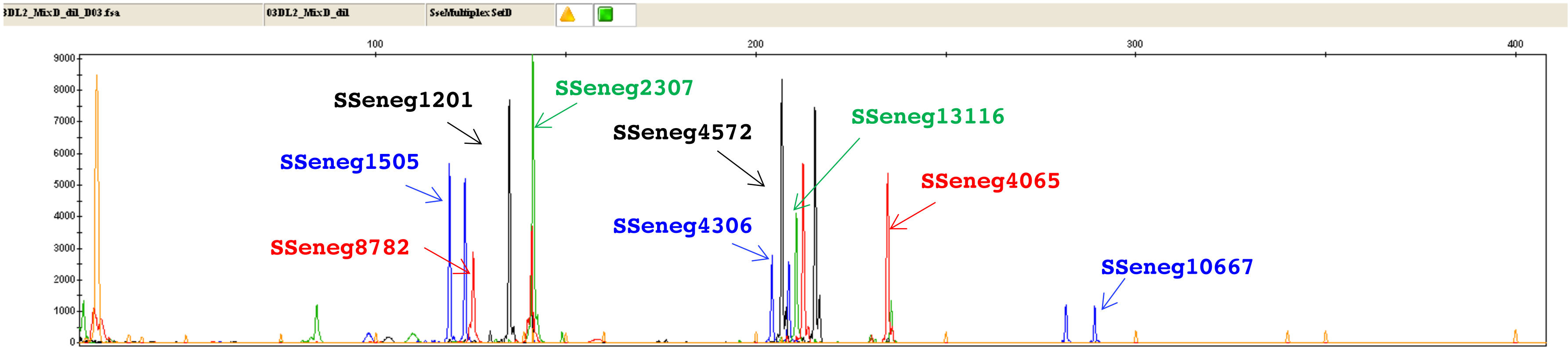

Multiplex E

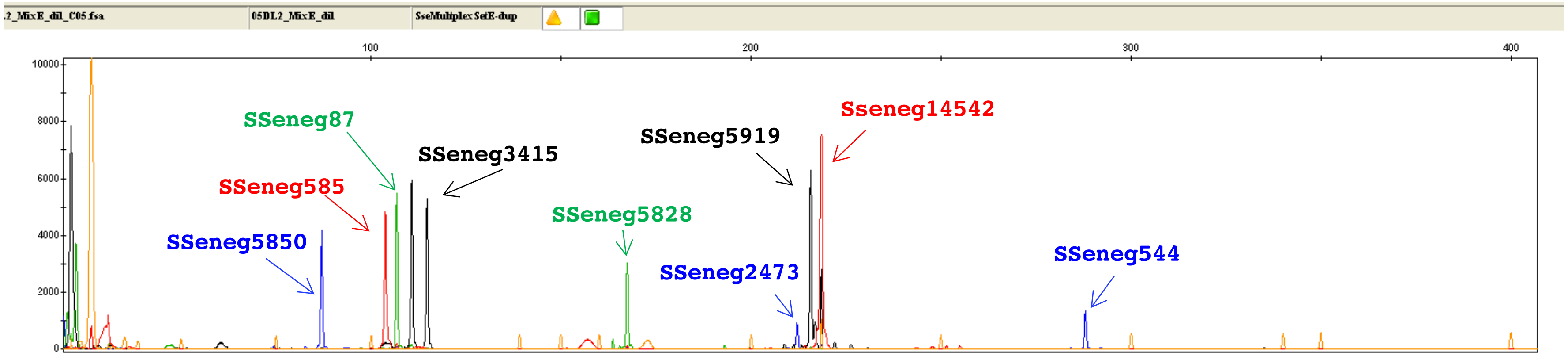

Multiplex F

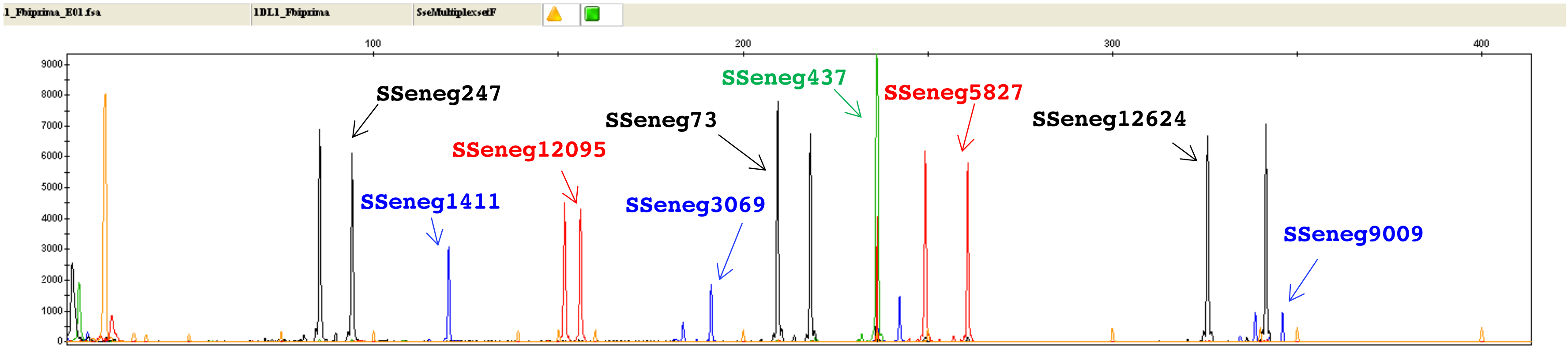

Multiplex G

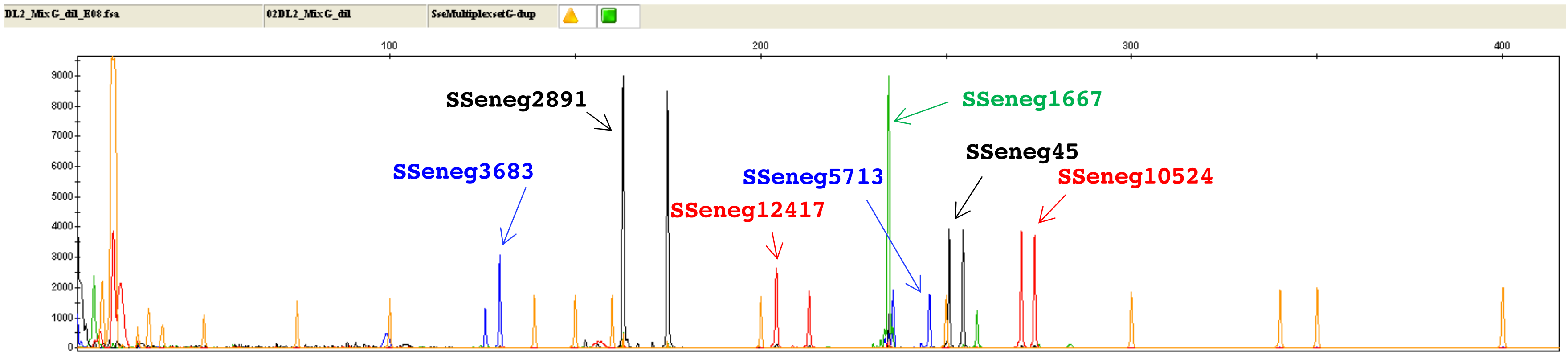

Multiplex H

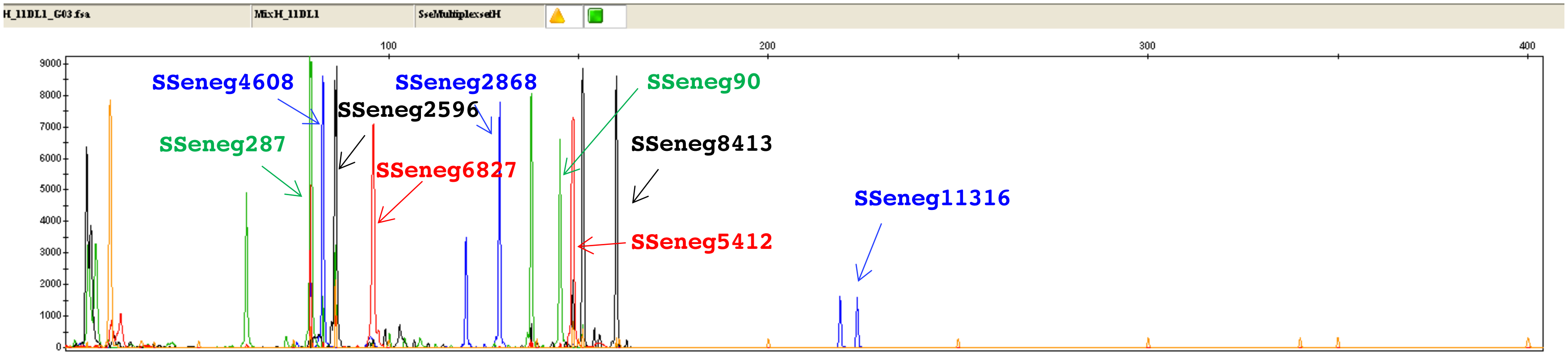

Multiplex I

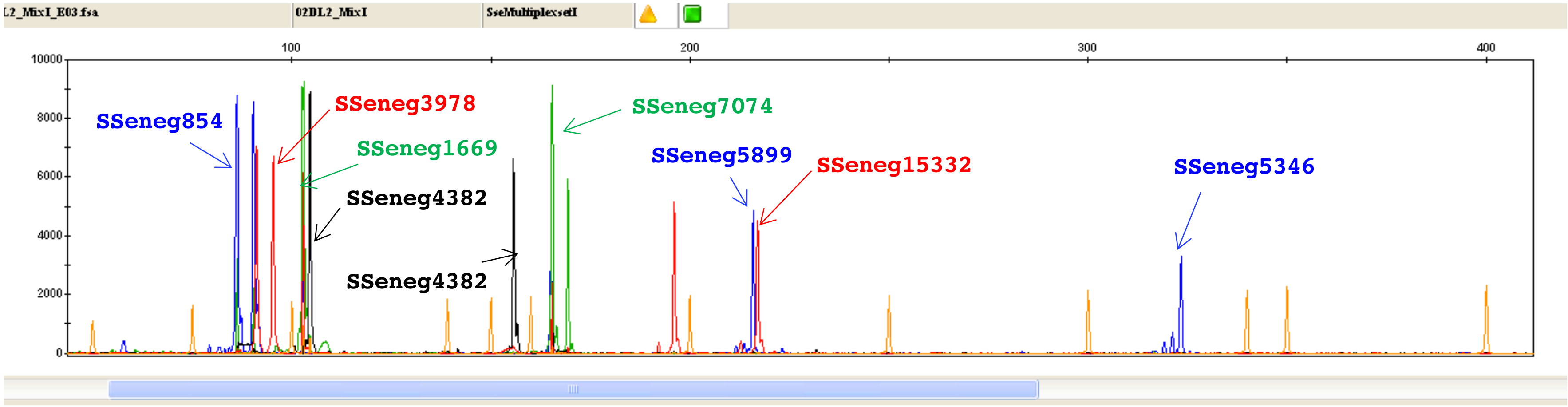

Multiplex J

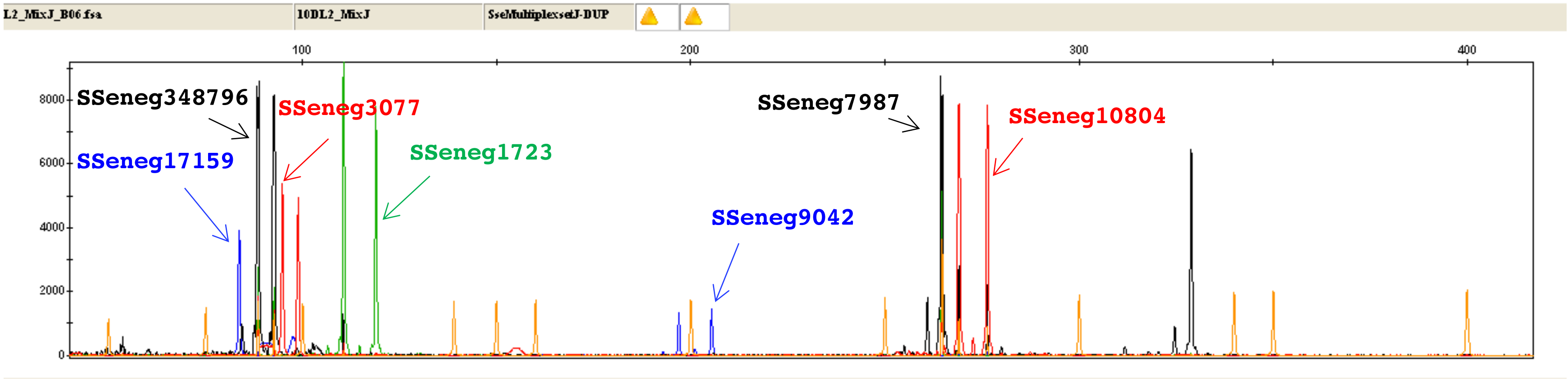

Multiplex K

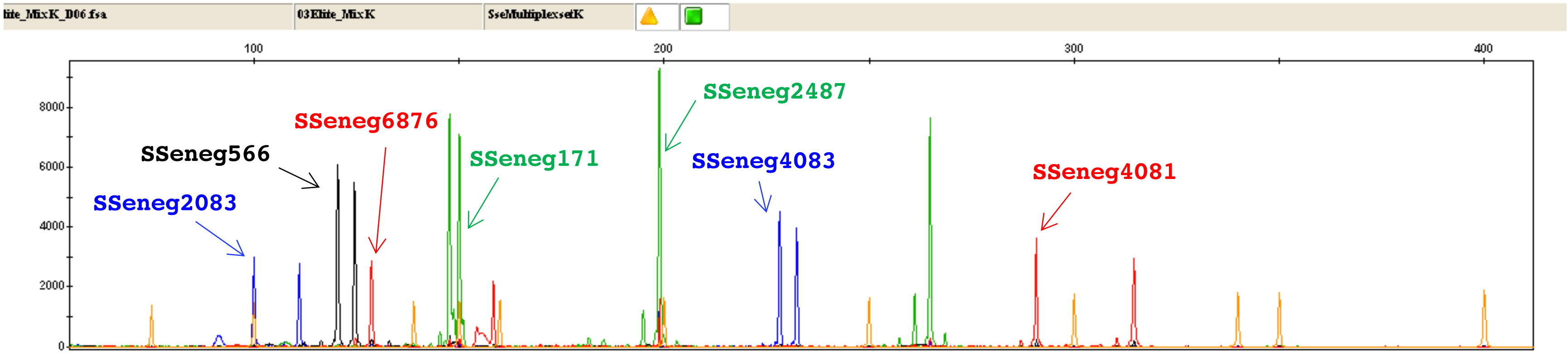

Multiplex L

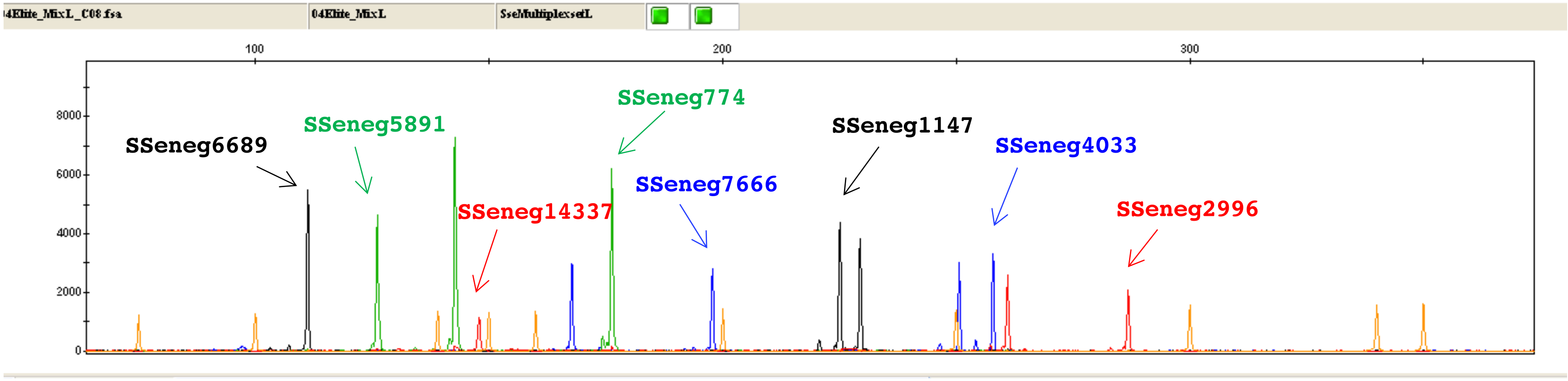

Multiplex M

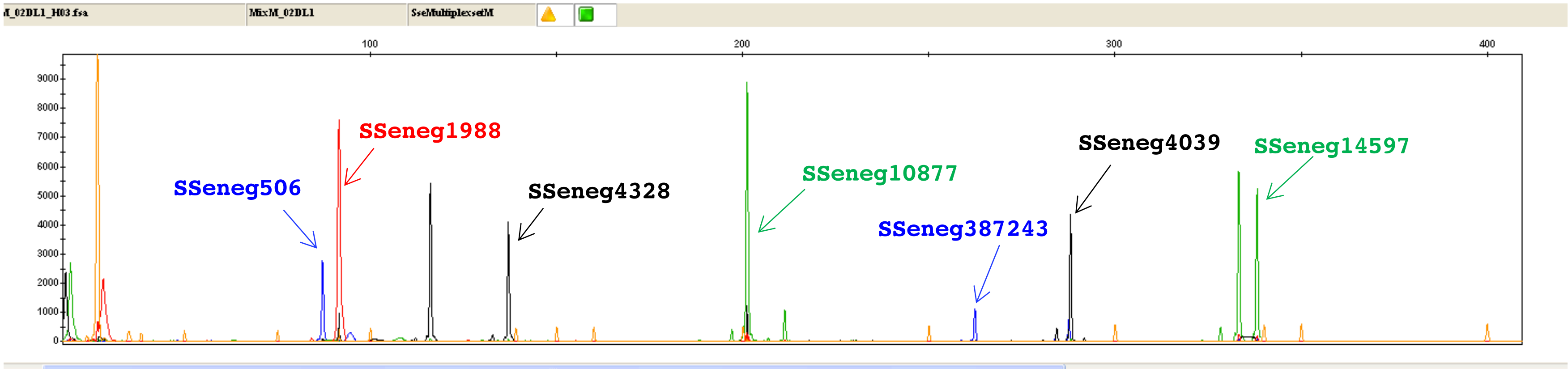

M_02DL1_H03.fsa
MixM_02DL1
SseMultiplexeM
100
200
300
400
9000
8000
7000
6000
5000
4000
3000
2000
1000
0
SSeneg506
SSeneg1988
SSeneg4328
SSeneg10877
SSeneg387243
SSeneg4039
SSeneg14597
