# Supplementary material for: Development of whole-genome multiplex assays and construction of an integrated genetic map using SSR markers in Senegalese sole
Source: Sci Rep. 2020 Dec 14;10:21905. doi: 10.1038/s41598-020-78397-w (PMC7736592; doi:10.1038/s41598-020-78397-w)
Supplement: Supplementary file 7 — Supplementary Information 7. [file 41598_2020_78397_MOESM7_ESM.docx]

|  | PIC | | Allelic range | |  |  |  |
| --- | --- | --- | --- | --- | --- | --- | --- |
| **SupermultiplexA** | **IFAPA** | **ULPGC** | **IFAPA** | **ULPGC** | **Type** | **C. semilaevis** |  |
| SSeneg4328 | 0.90 | 0.89 | 96-168 | 94-166 | Tetranucleotide | chr_1 | SseLG03 |
| SSeneg11269 | 0.95 | 0.93 | 183-263 | 183-263 | Dinucleotide | chr_5 | SseLG07 |
| SSeneg12624 | 0.82 | 0.79 | 311-359 | 327-355 | Pentanucleotide | chr_4 | SseLG08 |
| SSeneg2083 | 0.61 | 0.65 | 92-124 | 92-124 | Tetranucleotide | chr_16 | SseLG02 |
| SSeneg7666 | 0.90 | 0.85 | 162-224 | 162-222 | Dinucleotide | chr_19 | SseLG13 |
| SSeneg387243 | 0.85 | 0.83 | 250-316 | 252-304 | Tetranucleotide | chr_9 | SseLG06 |
| SSeneg4065 | 0.82 | 0.82 | 117-161 | 117-161 | Tetranucleotide | chr_11 | SseLG04 |
| SSeneg8782 | 0.81 | 0.76 | 200-242 | 200-240 | Tetranucleotide | chr_1 | SseLG16 |
| SSeneg4081 | 0.87 | 0.88 | 268-374 | 270-370 | Tetranucleotide | chr_13 | SseLG09 |
| SSeneg90 | 0.84 | 0.82 | 133-175 | 133-173 | Tetranucleotide | chr_17 | SseLG19 |
| SSeneg2487 | 0.93 | 0.92 | 188-328 | 188-312 | Tetranucleotide | chr_6 | SseLG10 |
| **SupermultiplexB** |  |  |  |  |  |  |  |
| SSeneg1201 | 0.81 | 0.83 | 115-180 | 105-180 | Pentanucleotide | chr_20 | SseLG01 |
| SSeneg5919 | 0.69 | 0.48 | 204-224 | 210-226 | Dinucleotide | chr_5 | SseLG12 |
| SSeneg4039 | 0.92 | 0.87 | 248-322 | 248-316 | Dinucleotide | chr_11 | Unplaced |
| SSeneg5772 | 0.78 | 0.57 | 80-130 | 94-114 | Tetranucleotide | chr_13 | Unplaced |
| SSeneg3069 | 0.85 | 0.65 | 183-245 | 193-245 | Tetranucleotide | chr_6 | SseLG10 |
| SSeneg4003 | 0.92 | 0.78 | 244-332 | 254-326 | Dinucleotide | chr_3 | SseLG01 |
| SSeneg12417 | 0.74 | 0.82 | 199-225 | 141-225 | Dinucleotide | chr_16 | SseLG02 |
| SSeneg10804 | 0.85 | 0.89 | 261-525 | 263-503 | Tetranucleotide | chr_z/w | SseLG05 |
| SSeneg1669 | 0.80 | 0.76 | 94-168 | 98-166 | Tetranucleotide | chr_9 | SseLG06 |
| SSeneg10877 | 0.77 | 0.75 | 177-223 | 179-223 | Tetranucleotide | chr_14 | SseLG21 |
| SSeneg14597 | 0.89 | 0.90 | 250-356 | 238-354 | Tetranucleotide | chr_3 | SseLG01 |
| SupermultiplexC |  |  |  |  |  |  |  |
| SSeneg433 | 0.57 | 0.55 | 101-174 | 103-123 | Tetranucleotide | chr_10 | SseLG11 |
| SSeneg7987 | 0.93 | 0.94 | 238-354 | 238-354 | Dinucleotide | chr_z/w | SseLG05 |
| SSeneg2868 | 0.80 | 0.81 | 112-172 | 106-138 | Tetranucleotide | chr_18 | SseLG20 |
| SSeneg5346 | 0.95 | 0.92 | 184-542 | 184-540 | Dinucleotide | chr_20 | SseLG01 |
| SSeneg10308 | 0.88 | 0.82 | 161-239 | 161-237 | Tetranucleotide | chr_11 | SseLG04 |
| SSeneg10524 | 0.66 | 0.65 | 266-286 | 250-290 | Tetranucleotide | chr_8 | SseLG18 |
| SSeneg87 | 0.62 | 0.76 | 106-166 | 96-160 | Tetranucleotide | chr_13 | SseLG09 |
| SSeneg6381 | 0.85 | 0.84 | 200-266 | 200-258 | Tetranucleotide | chr_9 | SseLG06 |
| **SupermultiplexD** |  |  |  |  |  |  |  |
| SSeneg53551 | 0.68 | 0.68 | 142-184 | 142-182 | Tetranucleotide | chr_6 | SseLG10 |
| SSeneg1973 | 0.92 | 0.87 | 249-329 | 249-325 | Tetranucleotide | chr_16 | SseLG02 |
| SSeneg4374 | 0.69 | 0.73 | 96-162 | 128- 160 | Tetranucleotide | chr_3 | SseLG01 |
| SSeneg5202 | 0.86 | 0.83 | 210-270 | 210-270 | Tetranucleotide | chr_8 | SseLG03 |
| SSeneg9009 | 0.92 | 0.90 | 286-368 | 272-356 | Tetranucleotide | chr_4 | SseLG08 |
| SSeneg6876 | 0.90 | 0.90 | 108-198 | 108-198 | Tetranucleotide | chr_20 | SseLG01 |
| SSeneg14542 | 0.71 | 0.72 | 202-244 | 202-244 | Tetranucleotide | chr_9 | SseLG06 |
| SSeneg2996 | 0.88 | 0.84 | 229-291 | 227-287 | Tetranucleotide | chr_10 | SseLG06 |
| SSeneg5891 | 0.75 | 0.74 | 97-159 | 111-175 | Tetranucleotide | chr_1 | SseLG16 |
| SSeneg1667 | 0.88 | 0.87 | 225-319 | 129-293 | Dinucleotide | chr_18 | SseLG20 |
